# Annexin A2 depletion exacerbates the intracerebral microhemorrhage induced by acute rickettsia and Ebola virus infections

**DOI:** 10.1371/journal.pntd.0007960

**Published:** 2020-07-20

**Authors:** Zhengchen Su, Qing Chang, Aleksandra Drelich, Thomas Shelite, Barbara Judy, Yakun Liu, Jie Xiao, Changchen Zhou, Xi He, Yang Jin, Tais Saito, Shaojun Tang, Lynn Soong, Maki Wakamiya, Xiang Fang, Alexander Bukreyev, Thomas Ksiazek, William K. Russell, Bin Gong

**Affiliations:** 1 Department of Pathology, University of Texas Medical Branch, Galveston, Texas, United States of America; 2 Department of Internal Medicine, Infectious Diseases, University of Texas Medical Branch, Galveston, Texas, United States of America; 3 Division of Pulmonary and Critical Care Medicine, Department of Medicine, Boston University Medical Campus, Boston, Massachusetts, United States of America; 4 Galveston National Laboratory, Galveston, Texas, United States of America; 5 Department of Neuroscience and Cell Biology, University of Texas Medical Branch, Galveston, Texas, United States of America; 6 Department of Microbiology and Immunology, University of Texas Medical Branch, Galveston, Texas, United States of America; 7 Department of Neurology, University of Texas Medical Branch, Galveston, Texas, United States of America; 8 Department of Biochemistry and Molecular Biology, University of Texas Medical Branch, Galveston, Texas, United States of America; Mahidol Univ, Fac Trop Med, THAILAND

## Abstract

Intracerebral microhemorrhages (CMHs) are small foci of hemorrhages in the cerebrum. Acute infections induced by some intracellular pathogens, including rickettsia, can result in CMHs. Annexin a2 (ANXA2) has been documented to play a functional role during intracellular bacterial adhesion. Here we report that *ANXA2*-knockout (KO) mice are more susceptible to CMHs in response to rickettsia and Ebola virus infections, suggesting an essential role of ANXA2 in protecting vascular integrity during these intracellular pathogen infections. Proteomic analysis via mass spectrometry of whole brain lysates and brain-derived endosomes from *ANXA2*-KO and wild-type (WT) mice post-infection with *R*. *australis* revealed that a variety of significant proteins were differentially expressed, and the follow-up function enrichment analysis had identified several relevant cell-cell junction functions. Immunohistology study confirmed that both infected WT and infected *ANXA2*-KO mice were subjected to adherens junctional protein (VE-cadherin) damages. However, key blood-brain barrier (BBB) components, tight junctional proteins ZO-1 and occludin, were disorganized in the brains from *R*. *australis*-infected *ANXA2*-KO mice, but not those of infected WT mice. Similar *ANXA2*-KO dependent CMHs and fragments of ZO-1 and occludin were also observed in Ebola virus-infected *ANXA2*-KO mice, but not found in infected WT mice. Overall, our study revealed a novel role of ANXA2 in the formation of CMHs during *R*. *australis* and Ebola virus infections; and the underlying mechanism is relevant to the role of ANXA2-regulated tight junctions and its role in stabilizing the BBB in these deadly infections.

## Author summary

Traditionally, spontaneous intracerebral microhemorrhages (CMHs) were defined as small foci of intracerebral hemorrhages. Such atraumatic CMHs are due to the rupture of a weak blood vessel wall. Infections complicating cerebrovascular accidents have been extensively investigated. However, the role of CMHs complicating infections, in particularly acute systemic infections, has been poorly explored. Population-based retrospective cohort studies suggest there are potentially more undiscovered cases of CMHs accompanying acute systemic infections. Given both the lack of an animal model and cellular/molecular pathophysiology of CMHs following acute systemic infections, there is an urgent need to increase our comprehensive understanding of acute infection-induced CMHs. Overall, our study revealed a novel role of annexin a2 (ANXA2) in the formation of CMHs during *R*. *australis* and Ebola virus infections; and the underlying mechanism is relevant to the role of ANXA2-regulated endothelial tight junctions and its role in stabilizing the blood-brain barrier in these deadly infections.

## Introduction

Vascular endothelial cells (ECs) are the common infection targets of rickettsia and Ebola virus.

Rickettsioses represent devastating human infections[1–7]. These arthropod-borne diseases are caused by obligatory intracellular bacteria of the genus *Rickettsia* (*R*.). A vaccine is not available for rickettsioses. Disseminated EC infection and EC barrier dysfunction are the central pathophysiologic features of human lethal spotted fever group rickettsial (SFGR) infections[8]. Typically, SFGR infection is controlled by appropriate broad-spectrum antibiotic therapy if diagnosed early. Nevertheless, SFGR infections present with nonspecific signs and symptoms rendering early clinical diagnosis difficult[7, 9]. Untreated or misdiagnosed SFGR infections are frequently associated with severe morbidity and mortality[1, 8, 10–12]. A fatality rate as high as 32% has been reported in hospitalized patients with Mediterranean spotted fever[12]. It has been forecasted that increased ambient temperatures under conditions of global climate change is a driver in rickettsial epidemiology, leading to more widespread distribution of rickettsioses[4]. Comprehensive understanding of rickettsial pathogenesis is urgently needed.

Ebola virus, a member of the family Filoviridae, causes severe Ebola virus disease in humans and nonhuman primates with case-fatality rates in humans of up to 90%[13–18]. Filoviruses target both the immune system[13, 19–25] and vascular endothelial cells (ECs), and cause a severe vascular leakage syndrome (VLS)[15, 26–29], but the underlying mechanisms remain unclear.

Spontaneous intracerebral microhemorrhages (CMHs) are defined as small foci of hemorrhages in the cerebrum [30–32]. These atraumatic CMHs are due to the rupture of small arteries, arterioles, and/or capillaries. CMHs have been a frequently recognized entity since the widespread application of magnetic resonance imaging[30, 33]. Recent investigations into CMHs have seen notable developments, and the increasing prevalence of CMHs is recognized as a significant problem [30, 31]. A population-based retrospective cohort study revealed that 18% of patients with central nervous system (CNS) infections developed CMHs within one year after the initial infection, 47 times greater than non-CNS infection controls[34]. Infections complicating cerebrovascular accidents have been extensively investigated[35–54]. However, the role of CMHs complicating infections[55–58], in particular, acute infections, has been poorly explored[34, 59–61]. To the best of our knowledge, four clinic reports from different countries described a total of 11 cases of CMH after acute systemic infections in patients ranging in age from 9 to 71 years[62–65]. Nine cases in two reports correlated the CMHs with specific pathogen infections, spotted fever (SF) rickettsiosis or ehrlichiosis[65]. Taken together, this information suggests there are potentially more undiscovered cases of CMHs accompanying acute systemic infections.

CMH can be acutely caused by pathogen-associated inflammation (AICMHs)[60, 66]. One of the underlying pathology of AICMHs is the acute dysfunction of the blood-brain barrier (BBB)[67] which is the interface between circulating blood and the central nervous system (CNS) and is composed of brain microvascular endothelial cells (BMECs), pericytes, and astrocytes[68, 69]. BBB properties are primarily determined by junctional complexes between the BMECs, i.e. adherens junctions (AJs) and tight junctions (TJs)[70–72]. Although ECs are susceptible to rickettsial and Ebola virus infections, the mechanisms of how the stability of the BBB is maintained during these infections is far from understood.

Annexin A2 (ANXA2) is a member of the large annexin family of Ca^2+^-regulated and phospholipid-binding proteins, which associates with cell membrane dynamics, cell-cell interactions, and cell adhesion[73–79]. ANXA2 can be monomeric, found mainly in the cytosol, or forming heterotetramer complex with S100A10 [75, 80]. S100A10 is a unique member of S100 protein family that has been known to bind to ANXA2. The interaction between ANXA2 and S100A10 yields a heterotetramer complex ANXA2-S100A10, enabling ANXA2 to translocate across the EC membrane and perform a variety of functions, facilitating plasmin-based fibrinolytic activities on vascular luminal surfaces[74, 75, 81]. Recently, we identified host ANXA2 as a novel receptor for SFGR and staphylococcus aureus adhesions to ECs[82]. However, there was no difference in rickettsial adhesion to or invasion into white blood cells between the wild-type (WT) and *ANXA2*-knockout (KO) mice.

Here we report an observation that focal CMHs lesions exist in the cerebra of *R*. *australis* infected *AXNA2*-KO mice but not *R*. *australis* infected WT mice. We hypothesize cell-cell junction in the BBB is destabilized in *AXNA2*-KO mice rendering them susceptible to AICMH. In order to comprehensively investigate this possibility, we performed a proteomic analysis using the whole brain lysate and brain-derived isolated endosomes. We identified a variety of differentially expressed (DE) proteins that were relevant to vascular integrity. Functional group annotation and network analysis based on the identified DE proteins revealed a variety of protein functional group changes, such as cell-cell junction, stress fiber, MHC II protein complex binding, and stress response. These identified functional groups support that a structural impairment of the BBB might be involved. Consistently, immunofluorescence (IF) of brain tissue of *R*. *australis* infected mice revealed dramatic disruption and disorganization of TJ proteins ZO-1 and occludin in *ANXA2*-KO mice, but not WT mice. We then chose to investigate whether a similar ANXA2 dependent disorganization of TJs is present in the context of Ebola virus, another pathogen known to attack ECs and present with severe vascular leakage. Interestingly, *ANXA2*-KO mice challenged by Ebola virus also exhibited CMHs and aberrant TJs whereas WT mice showed no signs of bleeding into CNS, indicating this pathology is not specific to rickettsia infection. Collectively, these data suggest that ANXA2 is required for the integrity of TJs in response to acute rickettsia and Ebola infections.

## Materials and methods

### Biologic containment

Infectious material and animals were handled in maximum-containment biosafety level 3 (for *R*. *australis*) and 4 (for Ebola virus) facilities at the Galveston Nationa Laboratory (GNL), University Texas Medical Branch at Galveston.

### Ethics statement

All animal protocols were approved by the Institutional Animal Care and Use Committee of the University of Texas Medical Branch (protocol # 1702018 and protocol # 9505045G). The animal studies were carried out in strict accordance with the recommendations in the Guide for the Care and Use of Laboratory Animals of the National Institutes of Health, USA.

### SFGR mouse infection model[83, 84]

*ANXA2*-KO on C57BL/6 background and C57BL/6 mice were used in this study. Animals (15 WT and 14 *ANXA2*-KO mice) were inoculated with 2LD50 dose (2 × 10^6^ pfu per mouse) of *R*. *australis* via tail vein injection and observed daily. All procedures followed the approved IACUC protocol. Signs of ruffled fur, hunched posture, labored breathing and closed eyelids were identified as lethal illness (41, 42). The animals were observed for 10 days when most of the animals were all in lethal illness state. For time-dependent pathological study, mice were inoculated with 2 LD50 dose of *R*. *australis* and euthanized at day 2, 4, 5 post-infection (n = 5 for each time point). For mass spectrometry experiments, animals (WT or *ANXA2*-KO) were euthanized at 5 days p.i. and the brain samples were collected and digested into protein lysate for downstream analysis. The time point was selected for LC/MS because this was when mice started to showing up lethal illness.

### Ebola virus mouse infection model[85]

To observe the effect of mice with *ANXA2*-KO compared to WT on Ebola hemorrhagic disease, C57BL6 6–12 week wild type (n = 5) and *ANXA2*-KO mice were inoculated intraperitoneally with 50 plaque-forming unit of mouse-adapted strain of Ebola Zaire (Mayinga) virus (provided by Thomas Ksiazek) in 200 uL of PBS. All procedures followed approved IACUC protocol. Mice were monitored multiple times daily for signs of illness and mortality p.i. Daily observations included evaluation of mice for clinical symptoms such as reduced grooming, ruffled fur, hunched posture, subdued response to stimulation, nasal discharge, and bleeding. Tissues and carcasses were collected for downstream assays.

### Sample collection

Blood samples were collected via orbital sinus and serum was obtained after centrifuge and discarding the cellular content of the blood. Complete necropsies were performed on all mice to obtain the organs. For immunohistochemistry or H&E staining, organs were fixed in a 4% (vol/vol) formaldehyde. For mass spectrometry, brain tissues were lysed in protein lysing buffer with proteinase inhibitor and phosphatase inhibitor. Endosome isolation was perform using Minute^TM^ endosome and cell fraction kit.

### IF and H&E staining

The fixed samples were subjected to H&E staining or immunofluorescence (IF) with an antibody against the protein of interest. Mouse tissues were collected and fixed in 10% formaldehyde solution for 72 hrs. Fixed tissues were washed 4–5 times with PBS. For antigen retrieval for IF studies, tissue sections were incubated in pH 6.0 citrate buffer and heated in a steamer (Black and Decker, New Britain, CT) for 10 minutes. After washing in PBS, samples were incubated with proteinase K for 5 minutes at RT. Tissue sections were first permeabilised by incubating with Triton X-100 (0.1% v/v in PBS) for 10 min. When using mouse monoclonal IgG against VE-cadherin (1/500) (Meridian Life Science, Saco, ME), tissues were blocked with unconjugated AffiniPure Fab fragment goat anti-mouse IgG (H+L) at 20 μg/ml(115-007-003, Jackson ImmunoResearch, PA) for 1 hour at room temperature. Tissue sections were incubated with rabbit antibodies against ZO-1 (1/500) (Thermo Fisher Scientific, Rockford, IL), occludin (1/500) (Thermo Fisher Scientific), SFG rickettsiae (1/2000) (provided by Dr. David Walker), or Ebola virus (1/500) (provided by Dr. Thomas Ksiazek) overnight at 4°C, followed by secondary antibody AlexaFluor 594 goat anti-mouse or AlexaFluor 488 or 594 goat anti-rabbit antibodies (1/1000) (Thermo Fisher Scientific). All tissue sections were followed by three final washes with PBS. Before mounting to the cover slide, the tissue sections were stained with DAPI. Normal rabbit and mouse IgGs were used as negative reagent controls (**S1C Fig**). Fluorescent images were taken with an Olympus BX51 microscope and analyzed using Olympus CellSens Standard software.

### DNA extraction and RT-qPCR

To quantify the rickettsia loading in the brain tissue, the DNeasy tissue kit (Qiagen, CA, 69506) was used to quantify DNA rickettsial DNA. Briefly, the brain samples were minced into pieces, and subjected to lysis buffer and proteinase k digestion for 10 minutes in 56 degrees Celsius; then DNA was precipitated in ethanol and purified using washing buffer. The purified DNA samples were stored in storage buffer in -20 C^o^. PCR was performed using the protocol as previously described [86]. Rickettsia-specific citrate synthase (CS) gene (gltA) was used as the target for rickettsia detection (gltA forward: GAGAGAAAATTATATCCAAATGTTGAT; gltA reverse, AGGGTCTTCGTGCATTTCTT)[86].

### ELISA

Plasma samples collected from *ANXA2*-KO and WT mice were used for ELISA to detect TNFα (Mouse TNFα Qantikine ELISA kit, MTA00B, R&D Systems; Minneapolis, MN) and IFNγ (Mouse IFNγ Qantikine ELISA, MIF00, R&D Systems). Standard curves were performed using the standard proteins according to the protocol provided in the ELISA kits. The ELISA plates were detected at 450nm.

### LC-MS/MS

Proteins were acetone-precipitated and cleaned with 1 ml of ice-cold wash solution (tri-*n*-butyl phosphate/acetone/methanol (1:12:1 by volume) for 90 minutes and then centrifuged at 2800g for 15 minutes at 4°C. The supernatant was removed and 1 ml ice-cold tri-*n*-butyl phosphate was added and incubated at 4°C for 15 minutes and then centrifuged at 2800g for 15 minutes at 4°C. The supernatant was discarded and 1 ml ice-cold acetone was added and incubated at 4°C for 15 minutes and then centrifuged at 2800g for 15 minutes at 4°C. The supernatant was discarded and 1 ml ice-cold methanol was added and incubated at 4°C for 15 minutes and then centrifuged at 2800g for 15 minutes at 4°C [87, 88]. 50ug of protein was solubilized with 5% SDS, 50 mM TEAB, pH 7.55, in the final volume of 25 uL. The sample was then centrifuged at 17,000g for 10 minutes for debris removal. Proteins were reduced by making the solution 20mM tris(2-carboxyethyl)phosphine TCEP (ThermoFisher, #77720) and incubated at 65^○^C for 30 minutes. The sample was cooled to room temperature. After 1 uL of 0.5 M iodoacetamide acid was added, the sample was allowed to react for 20 minutes in the dark. 2.75 ul of 12% phosphoric acid was added to the protein solution. 165uL of binding buffer (90% Methanol, 100mM TEAB final; pH 7.1) was then added to the solution. The resulting solution was loaded onto a S-Trap spin column (protifi.com) and passed through the column by a benchtop centrifuge (30-second spin at 4,000g). The spin column was washed with 400uL of binding buffer and centrifuged. The wash was repeated two more times. Trypsin was added to the protein mixture in a ratio of 1:25 in 50mM TEAB, pH = 8, and incubated at 37^○^C for 4 hours. Peptides were eluted with 80 uL of 50 mM TEAB, followed by 80 uL of 0.2% formic acid, and finally 80 uL of 50% acetonitrile, 0.2% formic acid. The combined peptide solution was then dried in a speed vac and resuspended in 2% acetonitrile, 0.1% formic acid, 97.9% water and placed in an autosampler vial[88].

### NanoLC MS/MS analysis

Instrument performance was verified by analyzing a standard peptide mix and a complex protein digest (HeLa) before the sample set was run between each experimental block and at the end of the experiment. The HeLa data files were analyzed in order to confirm that instrument performance remained consistent throughout the experiment. Peptide mixtures from digested brain tissue were analyzed by nanoflow liquid chromatography-tandem mass spectrometry (nanoLC-MS/MS) using a nano-LC chromatography system (UltiMate 3000 RSLCnano, Dionex), coupled on-line to a Thermo Orbitrap Fusion mass spectrometer (Thermo Fisher Scientific, San Jose, CA) through a nanospray ion source (Thermo Scientific). A trap and elute method was used. The trap column is a C18 PepMap100 (300um X 5mm, 5um particle size) from ThermoScientific. The analytical columns is an Acclaim PepMap 100 (75um X 25 cm) from (Thermo Scientific). After equilibrating the column in 98% solvent A (0.1% formic acid in water) and 2% solvent B (0.1% formic acid in acetonitrile (ACN)), the samples (1 μL in solvent A) were injected onto the trap column and subsequently eluted (400 nL/min) by gradient elution onto the C18 column as follows: isocratic at 2% B, 0–5 minutes; 2% to 32% B, 5–100 minutes; 32% to 50% B, 100–108 minutes; 50% to 90% B, 108–109 minutes; isocratic at 90% B, 109–114 minutes; 90% to 2%, 114–115 minutes; and isocratic at 2% B, till 130 minutes.

All LC-MS/MS data were acquired using XCalibur, version 2.1.0 (Thermo Fisher Scientific) in positive ion mode using a top speed data-dependent acquisition (DDA) method with a 3 sec cycle time. The survey scans (*m/z* 350–1500) were acquired in the Orbitrap at 120,000 resolution (at *m/z* = 400) in profile mode, with a maximum injection time of 50 msec and an AGC target of 400,000 ions. The S-lens RF level was set to 60. Isolation was performed in the quadrupole with a 1.6 Da isolation window, and CID MS/MS acquisition was performed in profile mode using rapid scan rate with detection in the orbitrap (res: 35,000), with the following settings: parent threshold = 5,000; collision energy = 35%; maximum injection time 100 msec; AGC target 500,000 ions. Monoisotopic precursor selection (MIPS) and charge state filtering were on, with charge states 2–6 included. Dynamic exclusion was used to remove selected precursor ions, with a +/- 10 ppm mass tolerance, for 60 sec after acquisition of one MS/MS spectrum.

Database Searching. Tandem mass spectra were extracted and charge state deconvoluted by Proteome Discoverer (Thermo Fisher, version 1.4.1.14). Deisotoping was not performed. All MS/MS spectra were searched using Sequest. Searches were performed with a parent ion tolerance of 5 ppm and a fragment ion tolerance of 0.60 Da. Trypsin was specified as the enzyme, allowing for two missed cleavages. Fixed modification of carbamidomethyl (C) and variable modifications of oxidation (M) and deamidation of asparagine and glutamine, were specified in Sequest. Scaffold (version Scaffold_4.8.7, Proteome Software Inc., Portland, OR) was used to validate MS/MS-based peptide and protein identifications. Peptide identifications were accepted if they could be established at greater than 95.0% probability. Peptide Probabilities from X! Tandem and Sequest were assigned by the Scaffold Local FDR algorithm. Peptide Probabilities were assigned by the Peptide Prophet algorithm [89] with Scaffold delta-mass correction. Protein identifications were accepted if they could be established at greater than 95.0% probability and contained at least 2 identified peptides. Protein probabilities were assigned by the Protein Prophet algorithm [89]. Proteins that contained similar peptides and could not be differentiated based on MS/MS analysis alone were grouped to satisfy the principles of parsimony.

### Functional enrichment analysis

**Fig 1:**
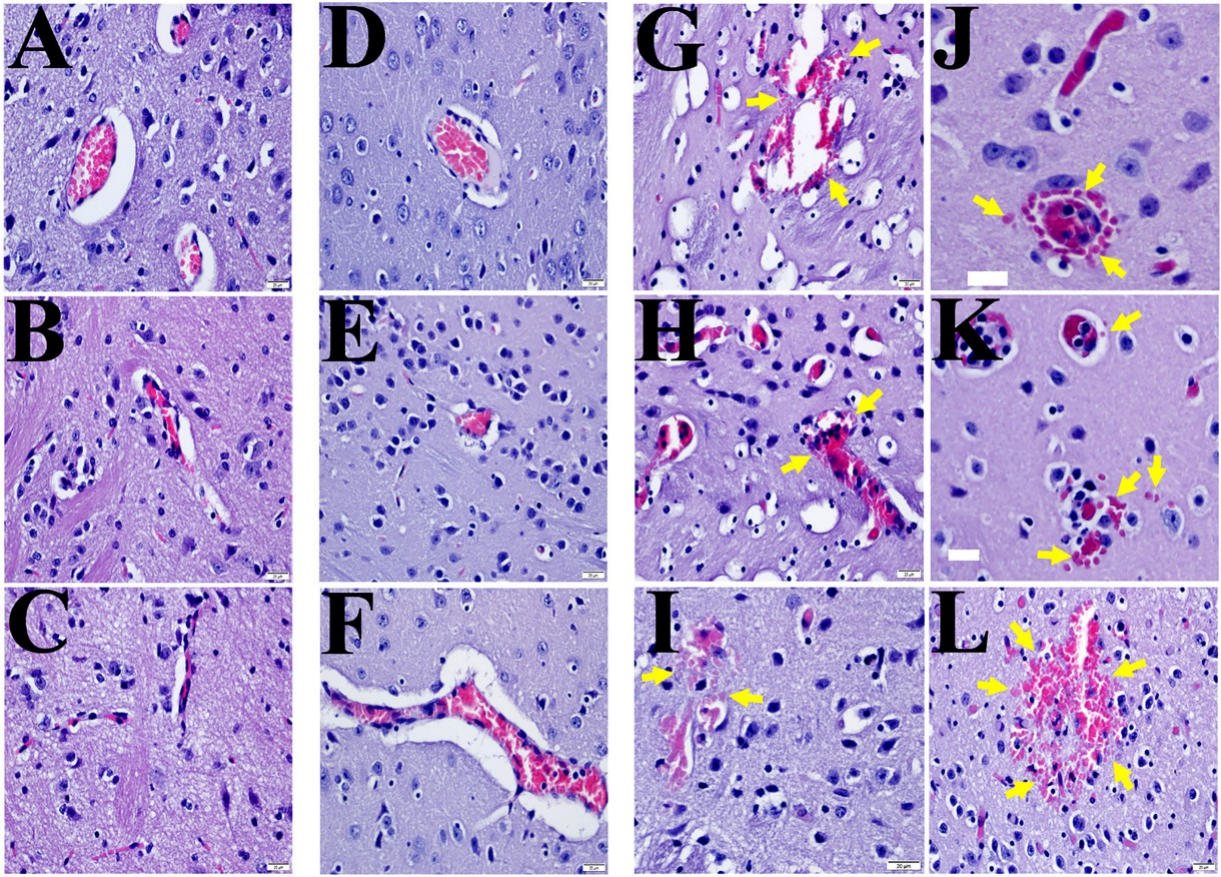
Representative H&E staining of the brains from *R*.*australlis*-infected *ANXA2*-KO and WT mice. Yellow arrows indicate the presence of focal hemorrhagic lesions. A, mock WT; B&C, mock *AXNA2*-KO; D-F, *R*. *australis*-infected WT; G-L), *R*. *australis*-infected *AXNA2*-KO mice. Scale bar: 20 μm.

Raw mass spectrometry data was filtered to rule out low abundance protein. Specifically, proteins that are included in the analysis must have at least 4 total spectra count. A list of differentially expressed proteins was obtained by comparing the -spectral counts between *ANXA2-*KO and WT groups and calculating the fold change. Proteins with a fold change greater than or equal to 2 were considered upregulated; protein with fold change less than or equal to -0.6 were considered down-regulated. Selected DE proteins are listed in Tables 1–4. Next, the DE proteins were further annotated using The Database for Annotation, Visualization and Integrated Discovery (DAVID) v6.8, which is also capable of doing functional enrichment analysis. Functional enrichment analysis helps us better correlate the molecular patterns with the gross pathology we have observed in the animal model. GO term and KEGG pathways were used to categorize the DE proteins (Fig 1). The Biological Networks Gene Ontology tool (BiNGO) [90] was used to perform the network analysis, which provides a direct structural visualization of the functional enriched groups. Protein-protein interaction (PPI) network was performed using Cytoscape GeneMania[91].

**Table 1:** Upregulated proteins and their KEGG pathway interpretation from whole-brain lysate. Fold change is calculated as (KO-WT)/WT.

Gene ID	Protein name	Fold change	KEGG Pathway
**AT2B1**	ATPase, Ca++ transporting, plasma membrane 1(Atp2b1)	4.17	Calcium signaling pathway, cGMP-PKG signaling pathway, cAMP signaling pathway, Adrenergic signaling in cardiomyocytes, Salivary secretion, Pancreatic secretion
**Q3UHH0, AT2B2**	ATPase, Ca++ transporting, plasma membrane 2(Atp2b2)	6.25	Calcium signaling pathway, cGMP-PKG signaling pathway, cAMP signaling pathway, Adrenergic signaling in cardiomyocytes, Salivary secretion, Pancreatic secretion
**VATA**	ATPase, H+ transporting, lysosomal V1 subunit A(Atp6v1a)	4.36	Oxidative phosphorylation, Metabolic pathways, Phagosome, Synaptic vesicle cycle, Collecting duct acid secretion, Rheumatoid arthritis
**AP1B1**	adaptor protein complex AP-1, beta 1 subunit(Ap1b1)	5	Lysosome
**CLH1**	clathrin, heavy polypeptide (Hc)(Cltc)	5.83	Lysosome, Endocytosis, Synaptic vesicle cycle, Endocrine and other factor-regulated calcium reabsorption, Huntington's disease, Bacterial invasion of epithelial cells
**ENOA**	enolase 1, alpha non-neuron(Eno1)	4.33	Glycolysis / Gluconeogenesis, Metabolic pathways, Biosynthesis of antibiotics, Carbon metabolism, Biosynthesis of amino acids, RNA degradation, HIF-1 signaling pathway
**HS90B**	heat shock protein 90 alpha (cytosolic), class B member 1(Hsp90ab1)	4.1	Protein processing in endoplasmic reticulum, PI3K-Akt signaling pathway, Antigen processing and presentation, NOD-like receptor signaling pathway, Progesterone-mediated oocyte maturation, Estrogen signaling pathway, Pathways in cancer, Prostate cancer
**HS90A**	heat shock protein 90, alpha (cytosolic), class A member 1(Hsp90aa1)	9.4	Protein processing in endoplasmic reticulum, PI3K-Akt signaling pathway, Antigen processing and presentation, NOD-like receptor signaling pathway, Progesterone-mediated oocyte maturation, Estrogen signaling pathway, Pathways in cancer, Prostate cancer
**A8DUK4**	hemoglobin, beta adult s chain(Hbb-bs)	4.09	African trypanosomiasis, Malaria
**H2B1B**	histone cluster 1, H2bb(Hist1h2bb)	4.67	Alcoholism, Viral carcinogenesis, Systemic lupus erythematosus
**H2B1K**	histone cluster 1, H2bk(Hist1h2bk)	4.67	Alcoholism, Viral carcinogenesis, Systemic lupus erythematosus
**Q8CBB6**	histone cluster 1, H2bq(Hist1h2bq)	4.67	Alcoholism, Viral carcinogenesis, Systemic lupus erythematosus
**MDHM**	malate dehydrogenase 2, NAD (mitochondrial)(Mdh2)	11.75	Citrate cycle (TCA cycle), Cysteine and methionine metabolism, Pyruvate metabolism, Glyoxylate and dicarboxylate metabolism, Metabolic pathways, Biosynthesis of antibiotics, Carbon metabolism
**PFKAM**	phosphofructokinase, muscle(Pfkm)	4.25	Glycolysis / Gluconeogenesis, Pentose phosphate pathway, Fructose and mannose metabolism, Galactose metabolism, Metabolic pathways, Biosynthesis of antibiotics
**KPYM**	pyruvate kinase, muscle(Pkm)	8.83	Glycolysis / Gluconeogenesis, Purine metabolism, Pyruvate metabolism, Metabolic pathways, Biosynthesis of antibiotics, Carbon metabolism, Biosynthesis of amino acids
**SNP25**	synaptosomal-associated protein 25(Snap25)	4.33	Synaptic vesicle cycle, Insulin secretion
**1433E**	tyrosine 3-monooxygenase/tryptophan 5-monooxygenase activation protein, epsilon polypeptide(Ywhae)	4.17	Cell cycle, Oocyte meiosis, PI3K-Akt signaling pathway, Hippo signaling pathway, Neurotrophin signaling pathway, Viral carcinogenesis

**Table 2:** Downregulated proteins and their KEGG pathway interpretation from whole-brain lysate. Fold change is calculated as (KO-WT)/WT.

Gene ID	Protein name	Fold change	KEGG Pathway
**A4GZ26, E9QAD8, D3Z5I6**	IQ motif and Sec7 domain 2(Iqsec2)	-0.63	Endocytosis
**D3YZU5**	SH3/ankyrin domain gene 1(Shank1)	-0.7	Glutamatergic synapse
**KCC2G**	calcium/calmodulin-dependent protein kinase II gamma(Camk2g)	-0.63	ErbB signaling pathway, Calcium signaling pathway, cAMP signaling pathway, HIF-1 signaling pathway
**E9Q1T1, A0A0G2JGS4**	calcium/calmodulin-dependent protein kinase II, delta(Camk2d)	-0.76	ErbB signaling pathway, Calcium signaling pathway, cAMP signaling pathway, HIF-1 signaling pathway
**MYH10, Q5SV64, Q3UH59**	myosin, heavy polypeptide 10, non-muscle(Myh10)	-0.78	Tight junction
**PCLO**	piccolo (presynaptic cytomatrix protein)(Pclo)	-0.76	Insulin secretion

**Table 3:** Upregulated proteins and their KEGG pathway interpretation from brain-derived endosomes. Fold change is calculated as (KO-WT)/WT.

Gene ID	Protein name	Fold Change	KEGG PATHWAY
**HMGCL**	3-hydroxy-3-methylglutaryl-Coenzyme A lyase(Hmgcl)	2	Synthesis and degradation of ketone bodies, Valine, leucine and isoleucine degradation, Butanoate metabolism, Metabolic pathways, Peroxisome
**L1CAM**	L1 cell adhesion molecule(L1cam)	2.5	Axon guidance, Cell adhesion molecules (CAMs)
**SHLB2**	SH3-domain GRB2-like endophilin B2(Sh3glb2)	2.33	Endocytosis
**AP2B1**	adaptor-related protein complex 2, beta 1 subunit(Ap2b1)	3.5	Endocytosis, Synaptic vesicle cycle, Endocrine and other factor-regulated calcium reabsorption, Huntington’s disease
**SYAC**	alanyl-tRNA synthetase(Aars)	2.17	Aminoacyl-tRNA biosynthesis
**PYGB**	brain glycogen phosphorylase(Pygb)	4.5	Starch and sucrose metabolism, Metabolic pathways, Insulin signaling pathway, Glucagon signaling pathway,Insulin resistance
**KCC4**	calcium/calmodulin-dependent protein kinase IV(Camk4)	2	Calcium signaling pathway, cAMP signaling pathway, Osteoclast differentiation, Long-term potentiation
**GPX1**	glutathione peroxidase 1(Gpx1)	3	Glutathione metabolism, Arachidonic acid metabolism, Thyroid hormone synthesis
**PGP**	phosphoglycolate phosphatase(Pgp)	3	Glyoxylate and dicarboxylate metabolism, Metabolic pathways, Biosynthesis of antibiotics, Carbon metabolism
**TLN1**	talin 1(Tln1)	3.33	Rap1 signaling pathway, Focal adhesion, Platelet activation, HTLV-I infection

**Table 4:** Downregulated proteins and their KEGG pathway interpretation from brain-derived endosomes. Fold change is calculated as (KO-WT)/WT.

ID	Gene Name	Fold change	KEGG PATHWAY
**CNDP2**	CNDP dipeptidase 2 (metallopeptidase M20 family)(Cndp2)	-0.6	Arginine and proline metabolism, Histidine metabolism, beta-Alanine metabolism, Metabolic pathways
**UGPA**	UDP-glucose pyrophosphorylase 2(Ugp2)	-0.75	Pentose and glucuronate interconversions, Galactose metabolism, Starch and sucrose metabolism, Amino sugar and nucleotide sugar metabolism, Metabolic pathways, Biosynthesis of antibiotics
**THIL**	acetyl-Coenzyme A acetyltransferase 1(Acat1)	-0.67	Fatty acid degradation, Synthesis and degradation of ketone bodies, Valine, leucine and isoleucine degradation, Lysine degradation, Tryptophan metabolism, Pyruvate metabolism
**AL7A1**	aldehyde dehydrogenase family 7, member A1(Aldh7a1)	-0.71	Glycolysis / Gluconeogenesis, Ascorbate and aldarate metabolism, Fatty acid degradation, Glycine, serine and threonine metabolism, Valine, leucine and isoleucine degradation, Lysine degradation
**BHMT1**	betaine-homocysteine methyltransferase(Bhmt)	-0.67	Glycine, serine and threonine metabolism, Cysteine and methionine metabolism, Metabolic pathways
**COF2**	cofilin 2, muscle(Cfl2)	-0.63	Axon guidance, Fc gamma R-mediated phagocytosis, Regulation of actin cytoskeleton,Pertussis,
**LGUL**	glyoxalase 1(Glo1)	-0.62	Pyruvate metabolism
**HSP7C**	heat shock protein 8(Hspa8)	-0.73	Spliceosome, MAPK signaling pathway, Protein processing in endoplasmic reticulum, Endocytosis, Antigen processing and presentation, Estrogen signaling pathway, Legionellosis, Toxoplasmosis, Measles, Influenza A
**HS90A**	heat shock protein 90, alpha (cytosolic), class A member 1(Hsp90aa1)	-0.63	Protein processing in endoplasmic reticulum, PI3K-Akt signaling pathway,Antigen processing and presentation, NOD-like receptor signaling pathway
**HPRT**	hypoxanthine guanine phosphoribosyl transferase(Hprt)	-0.67	Purine metabolism, Drug metabolism—other enzymes, Metabolic pathways,
**PDXK**	pyridoxal (pyridoxine, vitamin B6) kinase(Pdxk)	-0.78	Vitamin B6 metabolism, Metabolic pathways,
**KPYM**	pyruvate kinase, muscle(Pkm)	-0.67	Glycolysis / Gluconeogenesis, Purine metabolism, Pyruvate metabolism, Metabolic pathways, Biosynthesis of antibiotics, Carbon metabolism, Biosynthesis of amino acids,
**A1AT1**	serine (or cysteine) peptidase inhibitor, clade A, member 1A(Serpina1a)	-0.64	Complement and coagulation cascades
**A1AT2**	serine (or cysteine) preptidase inhibitor, clade A, member 1B(Serpina1b)	-0.64	Complement and coagulation cascades
**SYSC**	seryl-aminoacyl-tRNA synthetase(Sars)	-0.69	Aminoacyl-tRNA biosynthesis
**THOP1**	thimet oligopeptidase 1(Thop1)	-0.78	Renin-angiotensin system, African trypanosomiasis
**TKT**	transketolase(Tkt)	-0.83	Pentose phosphate pathway, Metabolic pathways, Biosynthesis of antibiotics, Carbon metabolism, Biosynthesis of amino acids

## Results

### Absence of ANXA2 is associated with the incidence of CMH in *R*. *australis* infection

First, we designed a survival study to investigate the potential role of ANXA2 in lethel *R*. *australis* infection. WT (n = 14) and *ANXA2*-KO (n = 15) mice were inoculated with an ordinarily lethal dose of *R*. *australis* (2 x 10^6^) via tail vein injection (i.v.) [82, 84] and observed up to 10 days post-infection (p.i.). Accumulative survival data were obtained from three independent experiments (**S1A Fig**) and subjected to Kaplan-Meier (K-M) analysis. We found no difference in survival between WT (21.43%) and *ANXA2*-KO (13.33%) groups. However, the gross pathology of the brain surface (**S1B Fig**) observed apparent different color cerebral areas between infected WT mice and infected *ANXA2*-KO. To examine underlying associated pathology, brain tissue sections were subjected to histological examination with hematoxylin and eosin (H&E) staining (**Fig 1**), which revealed striking focal CMHs, in the cerebra of all lethally infected *ANXA2*-KO mice (**Fig 1G–1L**), but not in the two surviving infected *ANXA2*-KO mice. Conversely, such CMHs were absent from both surviving and lethally-infected WT mice (**Fig 1D–1F**).

### Inactivation of ANXA2 does not affect the proliferation of *R*. *australis* or serum levels of IFNγ and TNFα in mice

**Fig 2:**
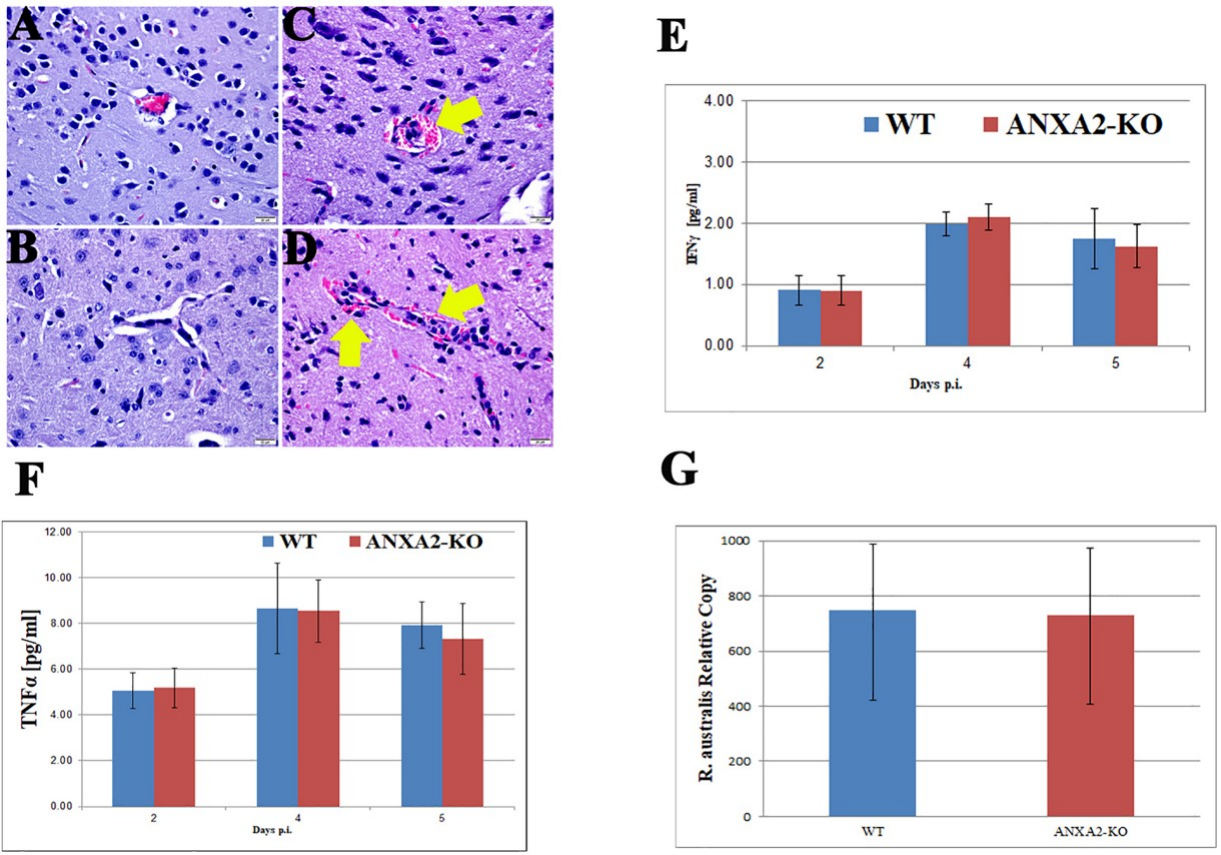
Representative H&E staining of brain sections from WT (A&B) and *ANXA2*-KO (C&D) 5 days post-*R*. *australis* infection. Perivascular hemorrhage (yellow arrow) can be observed in infected *ANXA2*-KO group but not infected WT group. TNFα (E) and IFNγ (F) concentrations in serum at 2,4,5 days post-*R*. *australis* infection. Relative R. australis DNA copies (G) extracted from the brain of WT and *ANXA2*-KO mice quantified by rt-qPCR. No significant difference was found. Error bar stands for standard deviation. Scale bar: 20 μm.

During the survival study, we observed mice becoming moribound at day 6 p.i. Therefore, we designed time-dependent pathological studies at days 2, 4, and 5 p.i. Mice were inoculated with an ordinarily lethal dose of *R*. *australis* (2 x 10^6^). At 5 days p.i. (5 mice per group), both *ANXA2*-KO and WT mice were euthanized as designed. H&E examination revealed extensive focal hemorrhagic lesions, at levels of arteriole, capillary, and venule, in the cerebra of all infected *ANXA2*-KO mice on day 5 p.i., but not WT mice (**Fig 2A–2D**).

**Fig 3:**
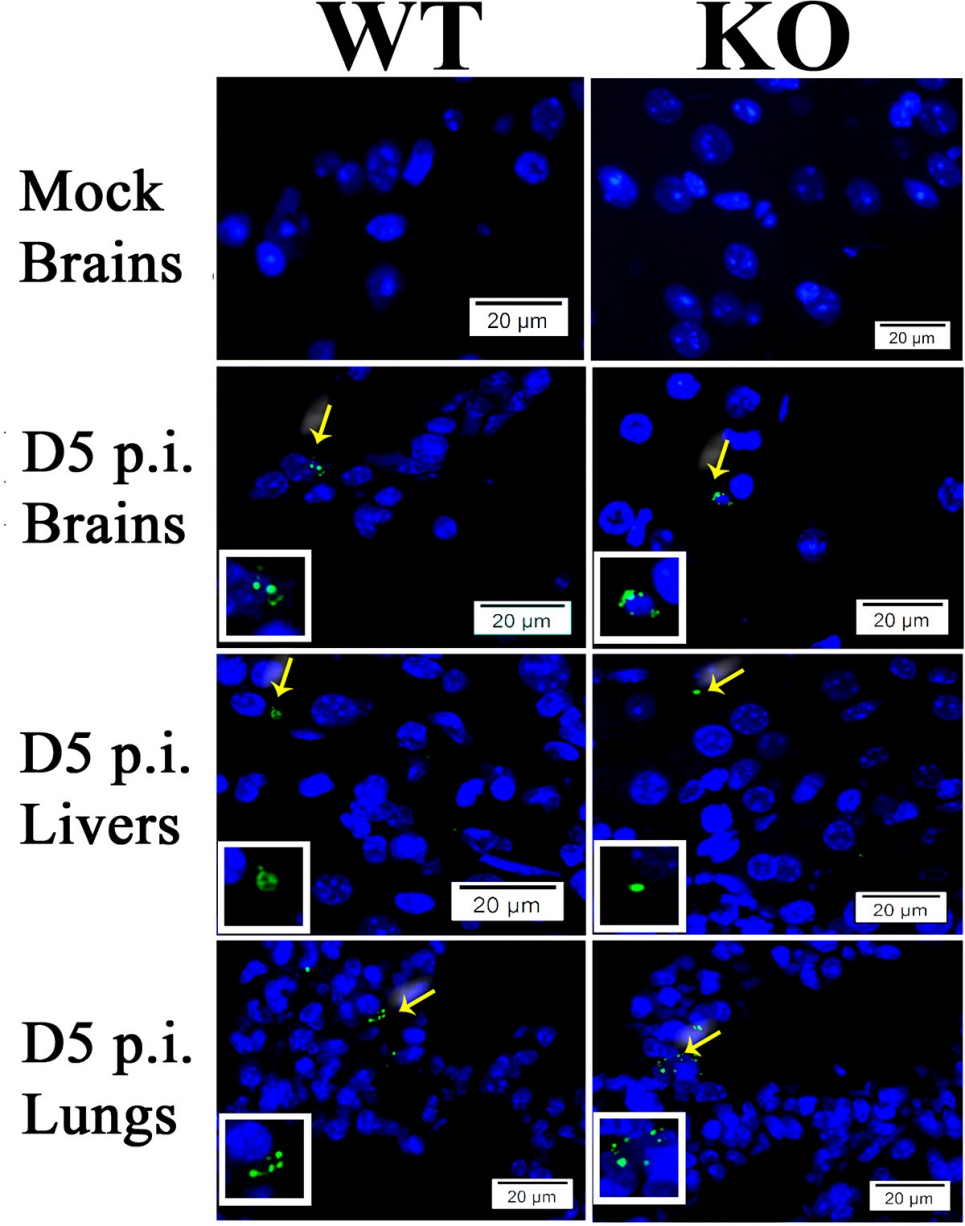
Representative IF staining of SFG rickettsiae (green) in livers, brains, and lungs from WT and *ANXA2*-KO mice with nuclei of host cells counter-stained with DAPI (blue). The areas indicated by the arrows are enlarged and distinguish rickettsial (green) staining (boxed inserts). Scale bars, 20 μm.

To examine whether ANXA2 plays a role in serum levels of IFNγ and TNFα in lethal *R*. *australis* infection, serum from mice at days 2, 4, or 5 p.i. were collected and proceeded to analyze the concentration of IFNγ and TNFα. However, no difference was found comparing WT and *ANXA2*-KO mice (**Fig 2E and 2F**). Meanwhile, on day 5 post-infection, the real-time qPCR analysis revealed no difference in bacterial loads in brain between WT (n = 4) and *AXNA2*-KO mice (n = 4) (**Fig 2G**). Immunofluorescent staining (IF) of rickettsia in the liver, brain and lung did not show any difference between WT and *ANXA2*-KO mice on day 5 p.i. (**Fig 3**). These data suggest *ANXA2* depletion does not affect the overall proliferation of *R*. *australis* or serum levels of IFNγ and TNFα in the mice.

### Proteomic analysis

In order to decipher the protein profile related to the CMHs observed in the *ANXA2-*KO mice post rickettsia infection, we performed a proteomic analysis of the whole brain protein lysate and isolated endosome. Isolated endosome protein pattern is important due to the involvement of ANXA2 in endocytosis and turnover of surface proteins and nucleotide[75].

#### Whole-brain lysate

**Fig 4:**
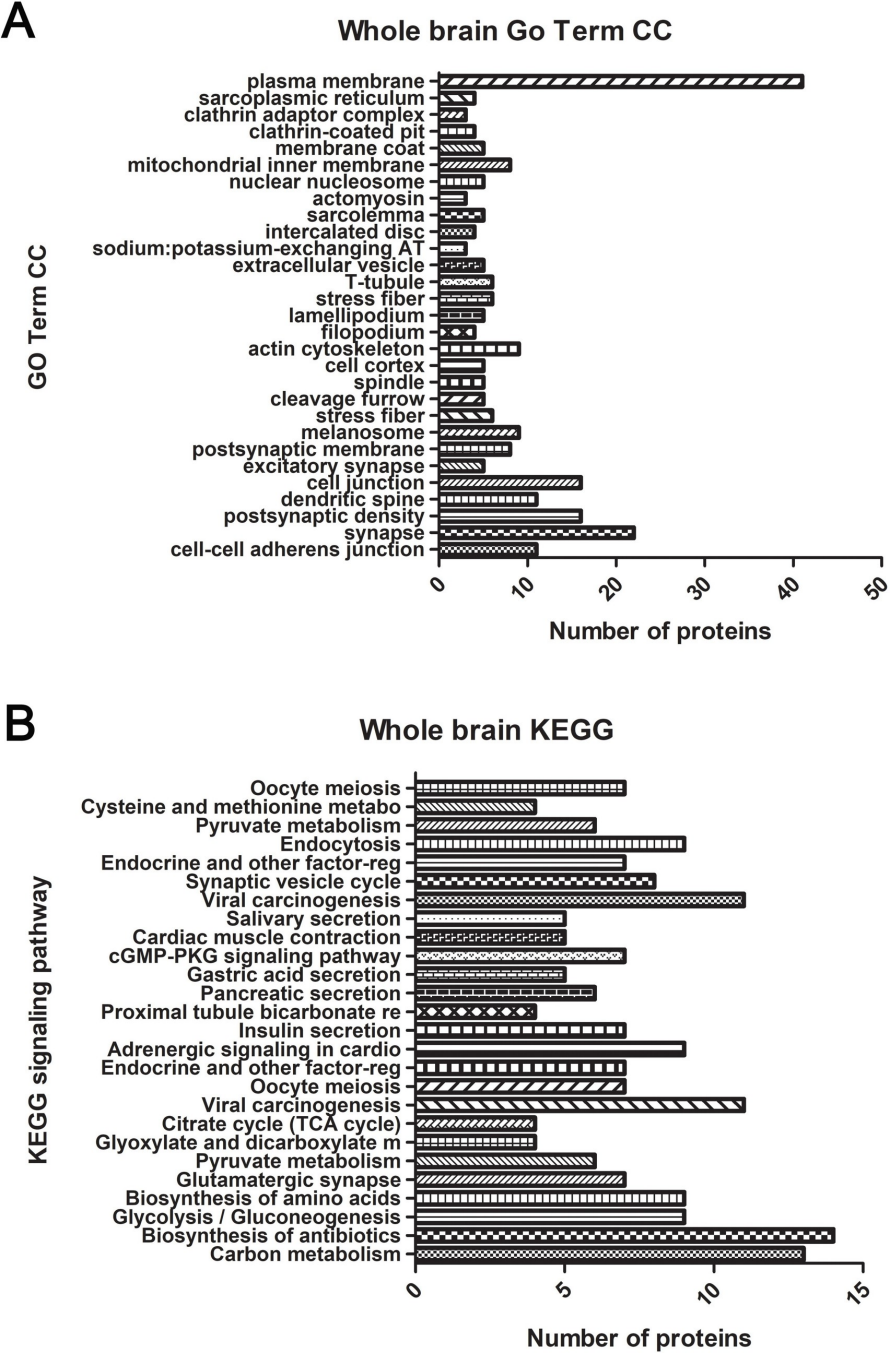
Functional annotations from GO Term CC (A) and KEGG (B) for whole-brain lysate. The gene lists are composed of differentially-expressed proteins from *ANXA2* KO and WT mice challenged with *R*. *australis* five days p.i. was analyzed using DAVID. Significantly enriched (p<0.05) functional GO term CC and KEGG pathways are listed. Y-axis indicates the significantly altered functional groups belonging to GO Term CC or KEGG pathways, x-axis indicates the number of the identified proteins in each category.

We compared the protein profiles of brain lysates from *ANXA2*-KO and WT mice challenged with *R*.*australis* on day 5 p.i., using LC/MS analysis, which identified one hundred thirty DE, with 93 upregulated and 37 downregulated (*ANXA2*-KO versus WT). The top upregulated and downregulated proteins are listed in **Tables 1 & 2**. It is noteworthy that the hemoglobin levels were higher (4-fold) in the infected *ANXA2-*KO mice compared to WT mice, which may account for the different gross pathology findings between the two groups.Functional enrichment analysis and visualization (**Fig 4 & S1 Table**) revealed statistically significant functional groups that are potentially associated with CMH in *ANXA2-*KO mice. The noteworthy upregulated proteins that are associated with the cell junction structure integrity include heat shock protein 90 α (HSP90α), heat shock protein 90 beta, enolase 1, clathrin (heavy polypeptide); important downregulated proteins include myosin (heavy polypeptide 10). Interestingly, TJ protein ZO-1 from the brain lysate is not differentially expressed in WT versus ANXA2 KO mice as measured by LC/MS.

Functional enrichment analysis was performed based on Gene Ontology (GO) Term and Kyoto Encyclopedia of Genes and Genomes (KEGG) pathway. Noteworthy GO Term include *cell-cell adherens junction*, *stress fiber*, *actin cytoskeleton*, *MHC class II protein complex binding*, *vesicle-mediated transport*, *and extracellular vesicle*. In KEGG pathway, we identified a variety of signaling pathways, in which *PI3K-Akt signaling pathway*, *cAMP signaling pathway*, *and protein digestion and absorption pathway* may be relevant to the pathology of CMHs.

#### Brain-derived isolated endosome

The endosome is integral to the endocytosis pathway. Due to the nature of endosomes, the proteins enriched in the endosomes are either subjected to degradation or recycled back to the plasma membrane[92]. Increased accumulation of proteins in the endosomal compartment suggests increased protein turnover rate and degradation; whereas reduction of protein enrichment in the endosomes might result in protein accumulation in another compartment such as cytosol or plasma membrane[92].

ANXA2 is known to be associated with the endosomal membrane. Endocytosis of several targets depends on the presence of tyrosine phosphorylation of ANXA2[93–98]. The absence of ANXA2 may lead to a differential endosomal protein profile, which may shed light on the underlying mechanisms associated with the increased susceptibility of *ANXA2*-KO mice to CMH upon rickettsia infection.

**Fig 5:**
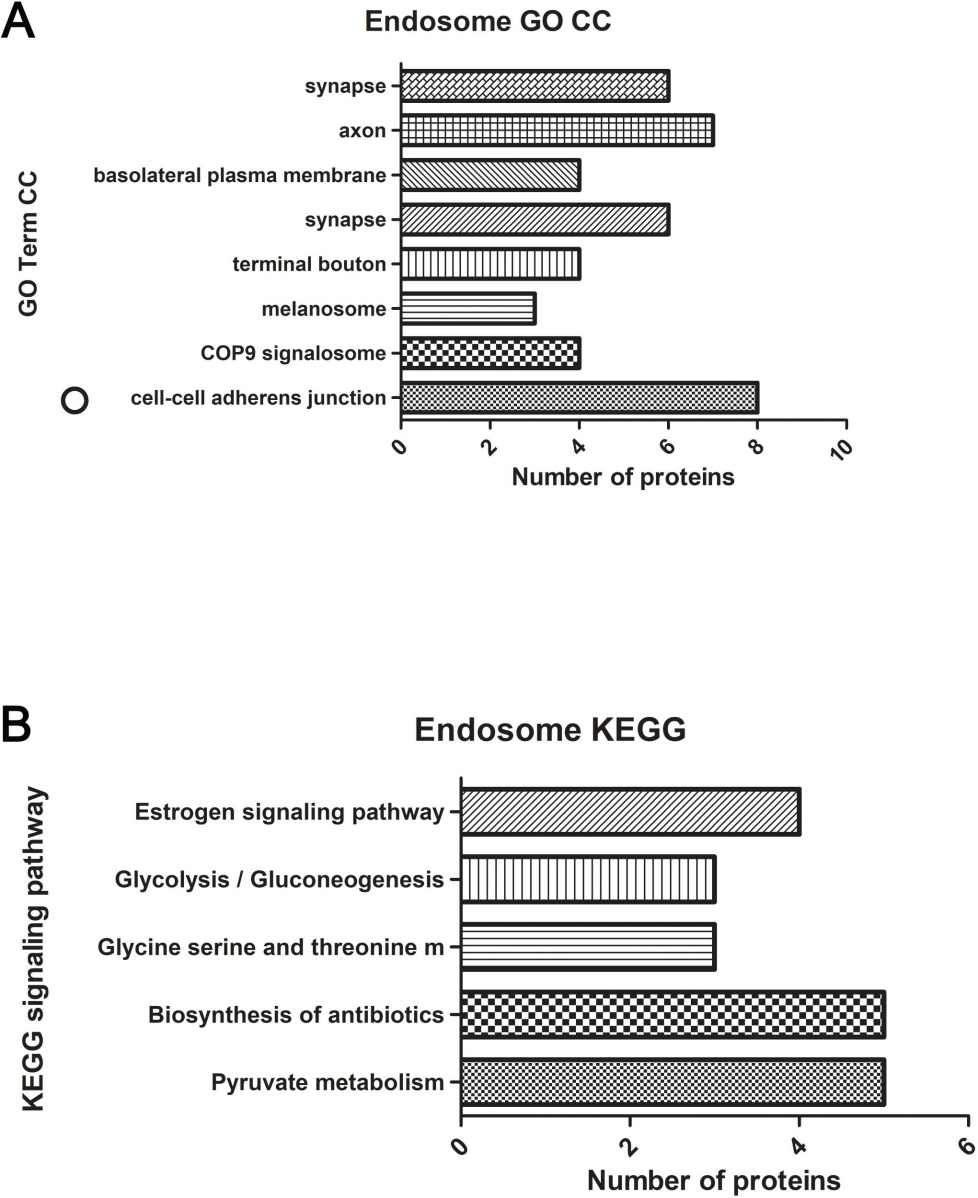
Functional annotation from Go Term CC (A) and KEGG (B) for isolated endosomes from the brain. Gene list composed of differentially expressed proteins comparing *ANXA2* KO and WT challenged by *R*. *australis* five days p.i. was analyzed using DAVID. Functional significantly (p<0.05) enriched GO term CC and KEGG pathways are listed. Y-axis indicates the significantly altered functional groups belonging to GO Term CC or KEGG pathways, and the X-axis indicates the number of the identified proteins in each category.

Comparing the endosomes isolated from the mouse brains of WT mice and *ANXA2*-KO 5 days after rickettsial infection, we have identified 47 DE proteins, with 18 upregulated proteins, and 29 downregulated proteins in the infected *ANXA2*-KO mice. Noteworthy upregulated DE proteins include talin1, alanyl-tRNA synthetase, glutathione peroxidase 1, and calcium/calmodulin-dependent protein kinase IV. Important downregulated proteins are cofilin 2, heat shock protein 8, HSP90α, thimet oligopeptidase and seryl-aminoacyl-tRNA synthetase. Top proteins are listed in **Tables 3 & 4**. Functional enrichment analysis was performed based on these 47 DE proteins, generating a list of functional enriched clusters (**Fig 5 & S1 Table**). Noteworthy Go Term functional groups include cell-cell AJ, metalloproteinase, and MHC II protein complex binding. Significantly altered signaling pathways in KEGG are pyruvate metabolism, biosynthesis of antibiotics, metabolic pathways, carbon metabolism, valine, leucine and isoleucine degradation, synthesis and degradation of ketone bodies.

**Fig 6:**
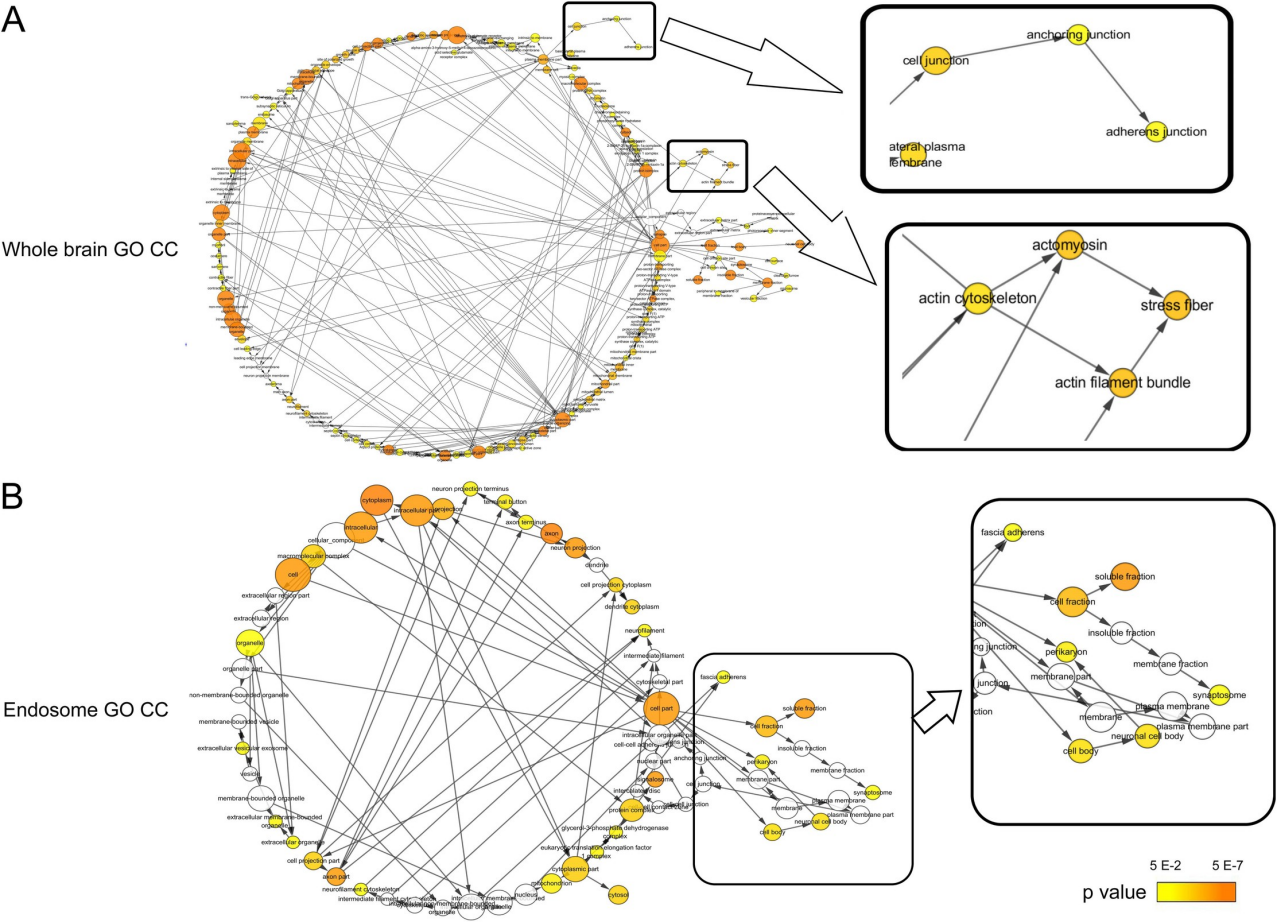
Visualization of the GO Term CC CC for whole brain lysate (A) and isolated endosome from the brain (B). Cytoscape BiNGO was used to generate the networks. Each node stands for a GO Term CC category, edges are present to connect functional related nodes. CMH-relevant functional groups (i.e., stress fiber and adherens junction) are enlarged and highlighted from the networks. The color bar indicates the range of p value corresponding to node (*ANXA2 KO* vs. WT in *R*. *australis* infection).

**Fig 7:**
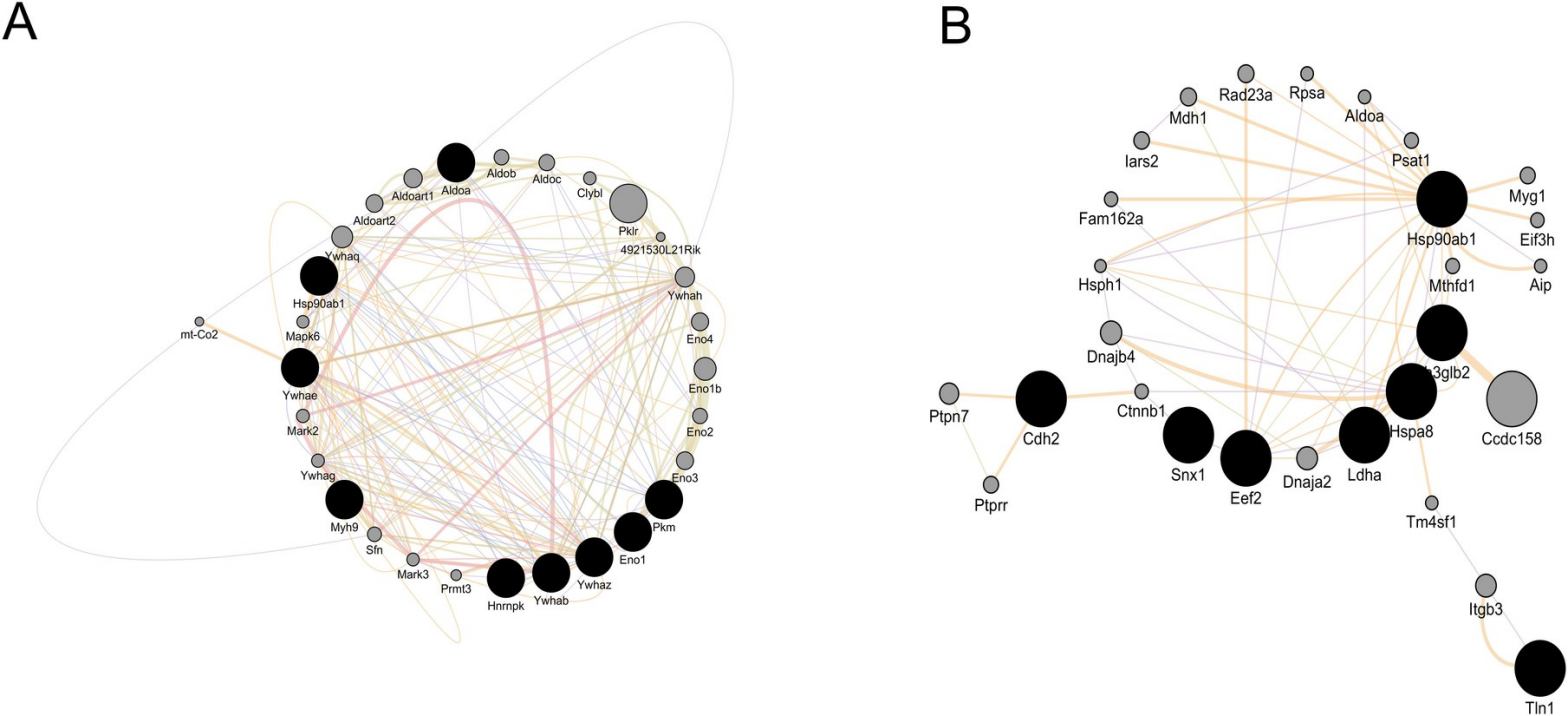
Cell junction associated protein-protein interactome from whole brain lysate (A) and brain derived isolated endosomes (B). Darker nodes are the source node, representing target genes associated with cell junctions. The gray nodes stands for the interacting genes for our target genes.

Whole brain lysate- and endosomal DE protein-derived functional enrichment (GO CC) network (**Fig** 6) analysis identifies the nodes associated with cell junction; and the interactome of the DE proteins under cell junction category is revealed in Fig 7A & 7B.

### Endothelial TJ proteins disorganized in brains of *ANXA2*-KO mice after lethal SFGR infections

The overall functional enrichment analysis supports our hypothesis that deletion of ANXA2 affects the cell-cell junction structure during the rickettsial infection. However, LC/MS showed that the expression level of TJ protein ZO-1 in the brain was similar between infected *ANXA2*-KO and infected WT mice. We postulated that strutures of TJ might be disorganized instead of downregulation of the expression levels.

**Fig 8:**
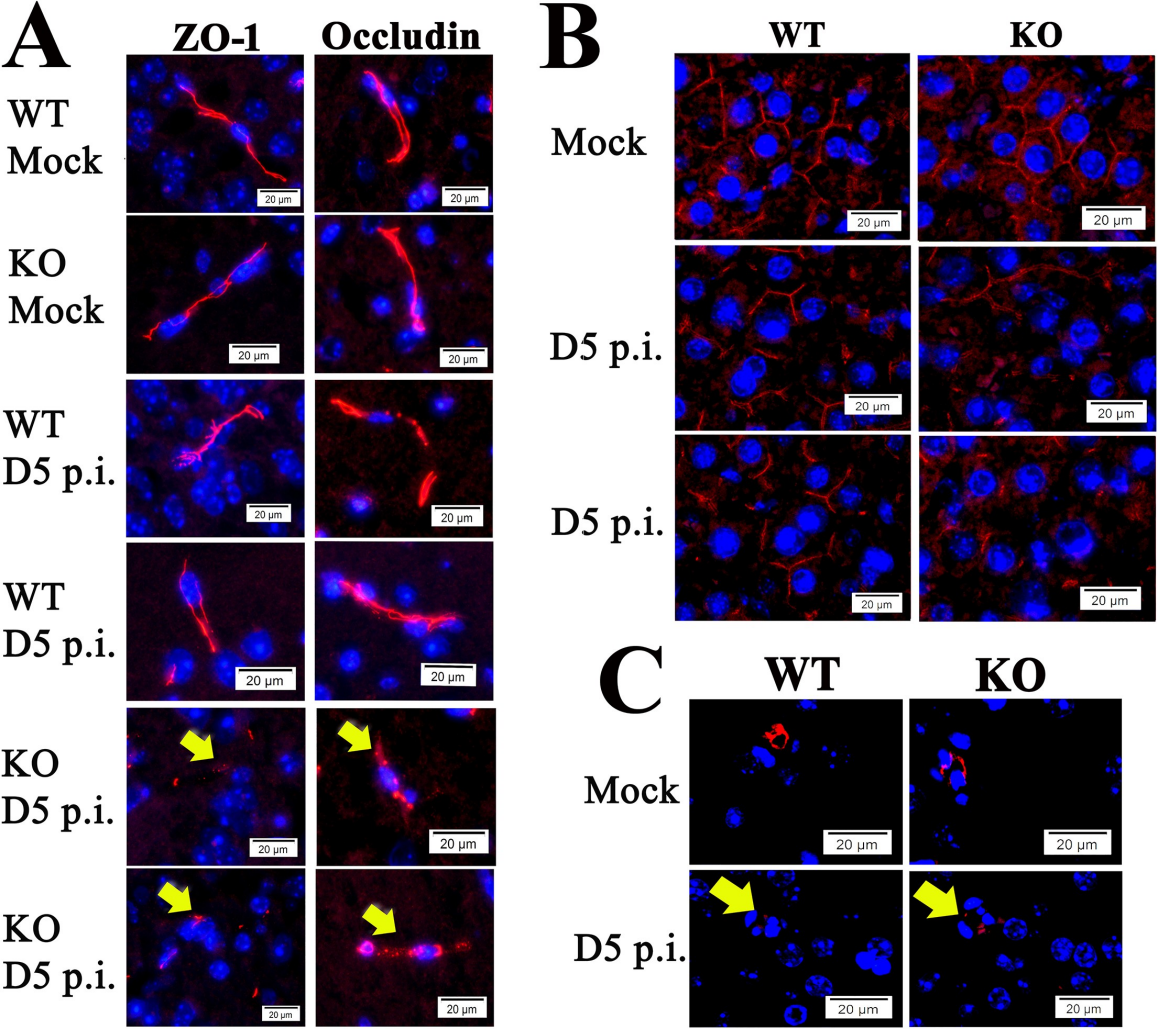
(A) Representative IF staining of ZO-1 (left) and occludin (right) in the brains from *R*.*australis*/mock-infected/ WT and *ANXA2*-KO mice, at day 5 p.i.. Yellow arrows indicate the fragmented structures of ZO-1 and occludin, which were mainly seen in infected *ANXA2*-KO mice. (B) Representative IF staining for occludin in the livers from *R*.*australis*/mock-infected/ WT and *ANXA2*-KO mice, at day 5 p.i.. (C) Representative IF for VE-cadherin in the brains from *R*.*australis*/mock-infected/ WT and *ANXA2*-KO mice, at day 5 p.i. Yellow arrows indicate the fragmented structures of VE-cadherin. Nuclei of mouse cells were counterstained with DAPI (blue). Scale bar: 20um.

Occludin is an important transmembrane protein integral to TJs, which has been previously shown to directly interact with ZO-1[99]. To investigate the structure of TJs, we applied IF assay to the brain tissue using antibody against ZO-1 and occludin. Remarkably, in all five *ANXA2-*KO mice, dramatic disruption and disorganization of ZO-1 and occludin were detected after D5 p.i. (**Fig 8A**). Interestingly, occludin disorganization was also observed in the livers of these *ANXA2*-KO mice on day 5 p.i. (**Fig 8B**). AJ protein VE-cadherins were also investigated. Animals in both WT and *ANXA2*-KO groups exhibited similar disintegrated structures of VE-cadherin in response to *R*. *australis* infection on day 5 p.i. (**Fig 8C**).

### CMHs and disorganized EC TJs in ANXA2-KO mice subjected to Ebola virus infection

**Fig 9:**
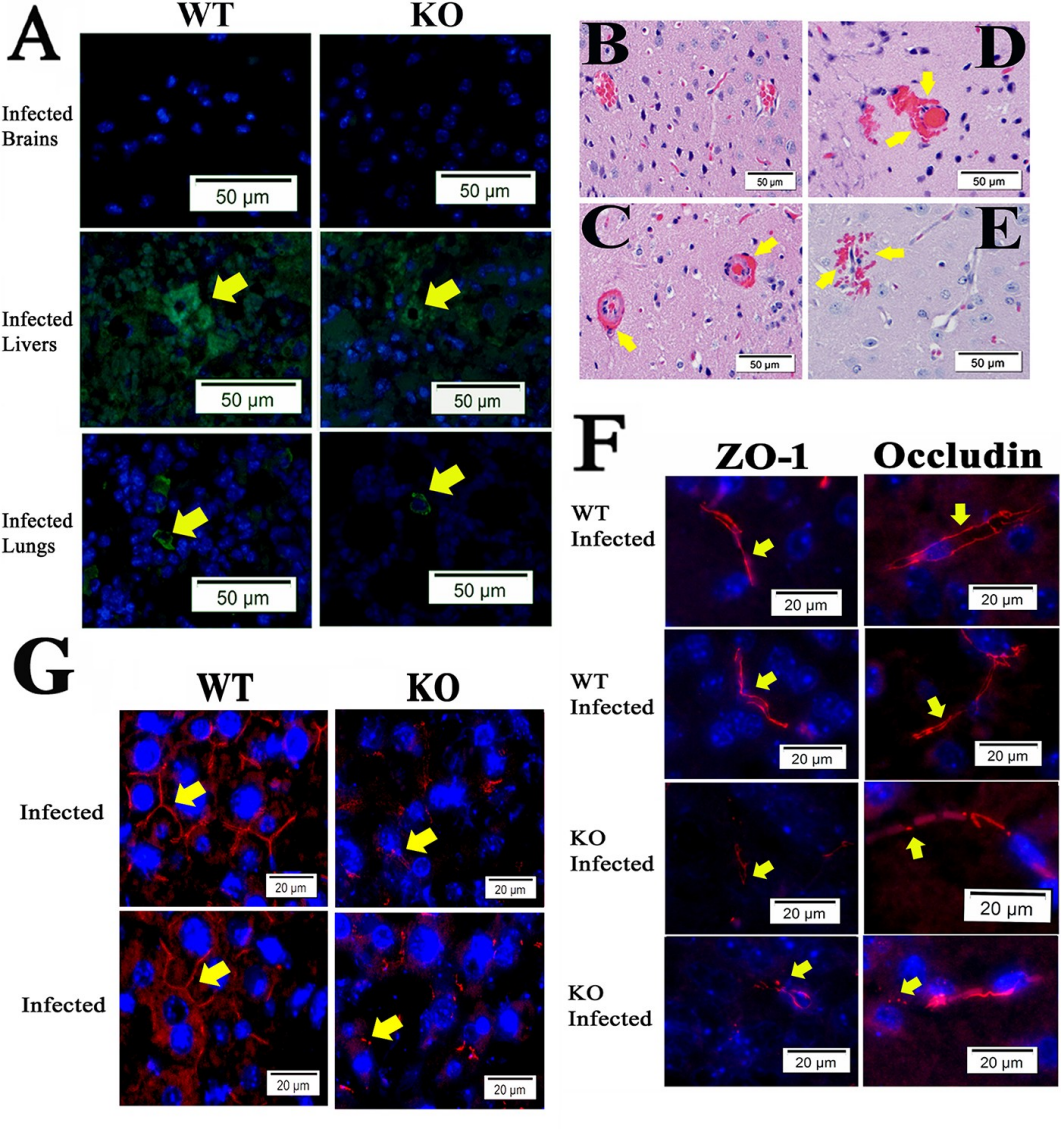
(A) Representative IF for Ebola virus antigen (Green) in the brains, livers, and lungs from WT or *ANXA2*-KO mice post-Ebola virus challenge. Yellow arrows represent the presence of Ebola virus antigen. (B-E) H&E staining of the brain from WT(B) and *ANXA2*-KO (C-E) mice infected by Ebola virus. Yellow arrows indicate the presence of perivascular hemorrhage. (F) Representative IF for ZO-1 (left) and Occludin (right) in the brains from WT or *ANXA2*-KO mice post-Ebola virus challenge. Yellow arrows indicate the position of ZO-1 or occludin, which represent the tight junction structure. *ANXA2*-KO but not WT Ebola virus-infected mice exhibited the fragmented tight junction structure in the brain. (G) Representative IF for occludin (red) in the livers from WT or *ANXA*2-KO mice day 10 post Ebola virus challenge. Yellow arrows show the structure of the pericellular occludin, which was relatively intact in Ebola virus-infected WT mice but dramatically fragmented in the Ebola virus-infected *ANXA2-KO* mice. Nuclei of mouse cells were counterstained with DAPI (blue). Scale bar: 50 μm (A-E), 20 μm (F and G).

We then chose to investigate whether a similar *ANXA2*-dependent disorganization of TJs is also present in Ebola infection, given that both Ebola virus and rickettsiae are known to attack endothelial cells and disease progression is marked by severe vascular leakage[15, 26–29, 100]. We first investigated whether the *ANXA2* deletion-dependent CMHs could be observedin the context of Ebola virus infection via retrospective histology examination of brain tissue samples from WT (n = 5) or *ANXA2*-KO (n = 5) mice challenged by a mouse-adapted strain of Ebola Zaire virus (50 plaque-forming unit by the intraperitoneal route[85]). In this experiment, a 20% cumulative survival was observed in *ANXA2*-KO mice 12 days p.i., compared with an 80% cumulative survival in the group of WT mice at the same time (p = 0.08, Log-rank test) (**S2 Fig**). IF assay of Ebola virus in the liver, brain and lung tissues showed no difference between WT and *ANXA2*-KO mice on day 7–12 p.i. (**Fig 9A**). The sample size was not robust enough to allow a conclusion regarding survival between groups, as this was not the goal of the study. However, future studies comparing the survuival between WT and *ANXA2*-KO mice infected with Ebola virus using an increased number of mice in each group are planned.

Similarly, CMHs were detected using H&E staining in all *ANXA2*-KO mice (**Fig 9C–9E**), but not in WT mice (**Fig 9B**) during 7–12 days p.i. Disorganized TJ protein ZO-1 and occludin were also present in brain tissue from *ANXA2*-KO, but not WT mice (**Fig 9F**) infected with same dose of Ebola virus intraperitoneally. Furthermore, dramatic disruption and disorganization of occludin were also observed in the livers of *ANXA2*-KO mice post Ebola virus infection (**Fig 9G**).

Collectively, these data suggest disorganization ofthe TJ structures as an underlying mechanism of the CMHs observed in *ANXA2*-KO mice post-*R*. *australis* or Ebola virus infections.

## Discussion

In this study, we report a novel finding that *ANXA2*-KO mice are more susceptible to rickettsia- or Ebola virus-induced CMHs, underlying mechanisms relevant to endothelial TJs-based BBB dysfunction. Furthermore, we performed a proteomic analysis on the whole brain lysate and isolated endosomes using LC/MS. The obtained list of differentially expressed proteins was subjected to function enrichment analysis, combining GO term and KEGG pathway.

### Whole-brain lysate proteomic functional group

#### Cell-cell junction

The impermeability properties of BBB are primarily determined by BMEC AJs and TJs, which offer contracting forces to bridge the adjacent ECs via cadherins and nectins [101]. AJs directly and indirectly via via actin-associated proteins interact with TJs, together they grant the essential stability of the BBB.[99, 101]. AXNA2 affects the stability of AJs. The depletion of ANXA2 has been shown to dissociate VE-cadherin from AJ[76]. The DE proteins specifically under AJs category include myosin heavy polypeptide 9 (non-muscle), septins, tyrosine 3-monooxygenase/tryptophan 5-monooxygenase activation protein, HSP90α (cytosolic), aldolase A, enolase 1, heat shock protein 5.These proteins are not direct structural proteins of the AJs such as VE-cadherin and beta-catenin but contribute to developmental AJs assembly and. For example, inhibition of HSP90α is capable of attenuate the reduced VE-cadherin and beta catenin level induced by thrombin, LPS, VEGF, or TGF-β1, which all are known to increase endothelial permeability [102–104]. It is also suggested that HSP90α is a downstream effector of RhoA signaling mediated by LPS[104]. Our LC/MS analysisrevealed a 9.4 fold increase in HSP90α in *ANXA2*-KO mice (whole brain lysate) compared to WT 5 days p.i. . . Elevated levels of HSP90α could lead to the instability of the AJ in the presence of LPS[105]. There is only one published study supporting the association, showing that high glucose increases *ANXA2* expresasion in ECs and enhances the association of HSP90α and ANXA2 in both ECs and rat aorta[106]. The functional interaction between ANXA2 and hsp90 in the context of pathogen infection is largely unknown.

#### Stress fiber

Stress fiber is formed by contractile actin filament bundle and a variety of proteins responsible for actin stabilization including septins, α-actin, tropomyosin, Rho-associated protein kinase (ROCK), fascin, and myosin 2, etc.[107]. It is formed in the cells in response to the environmental mechanical stress, such as ECs in the presence of fluid shear stress. Cells cultured on stiff substrate form stress fiber whereas those on the soft substrate have much less fiber. Physiologically, stress fiber is important for cell adhesion, mechanotransduction and AJ maturation[107–109]. Stress fiber plays a positive role in AJ maturation, but it is not always the case in the pathological environment. Such phenomenon is also seen in VEGF- and activated-neutrophil-treated ECs[110, 111].

Our data report four DE proteins in the stress fiber category: septin 5, septin 7, tropomyosin 1 and tropomyosin 3. Three out of the four proteins were increased in *ANXA2*-KO group. The association of ANXA2-dependent integrity of TJs to stress fiber formation is elusive. One of the possibilities is relevant to the dynamic between cortical actin cytoskeleton and stress fibers. TJs, the core structures of the BBB that seal BMECs, are at the most apical of all junctional components[101, 112]. ZO-1 has been identified as a functional adaptor to link other TJ components to the cortical actin cytoskeleton[113], which lies just beneath the plasma membrane. When stress fibers are formed in response to different stimuli, such as inflammation and oxidative stress, cortical actins are lost, potentially causing disturbances in the TJ aperatus[114, 115]. In BHK-IR cells, insulin-mediated loss of stress fiber and resulting cell detachment depend on the presence of AXNA2 and its tyrosine phosphorylation state[77]. Silencing *ANXA2* with siRNA in MOI Muller cells directly leads to the accumulation of stress fiber[73].

### Isolated endosome function group

#### Cell-cell junctions

AJ is a significantly enriched functional group. Ten proteins were found in this category. Endocytosis is known to mediate the stability of AJ[116]. Based on structural analysis using IF and drug intervention, Georgiou et al., suggested that impaired endocytosis leads to AJ instability[116]. AXNA2 is involved in the formation of early endosomes. AXNA2 depletion results in morphological changes in endosomes and alters their distribution[76]. Our proteomic data analyzing the protein difference between WT and *ANXA2*-KO mice upon rickettsial infection identified 10 proteins associated with AJ in the brain endosomal compartment. Interestingly, cadherin complex protein cadherin 2 and talin 1 were upregulated in *ANXA2*-KO after infections. Cadherin 2 is a direct structural protein of AJ, and it is involved in pericyte-EC adhesion, which is critical for the integrity of the BBB. Focal injection of antibody against cadherin-2 is sufficient to induce intracerebral hemorrhage[117]. The biological significance of cadherin-2 upregulation in the endosomal compartment is unknown. It has been shown, however, that cadherin-2 and other several other junction-associated proteins were subjected to rapid endocytosis in a drug-induced disruption of cell-cell contact model [118]. Talin 1 is a well-known AJ-associated protein located at the cytoplasmic side to the AJ[119]. Endosomal talin has been shown to regulate the function of endosomal integrin[120].

#### MHC II protein complex binding and stress response

Three major players are found in MHC II protein complex binding: HSP90α, heat shock protein 8 (HSPa8), and pyruvate kinase. HSP90α is noteworthy. In *ANXA2-*KO mice, it was drastically enriched in whole-brain but decreased in the endosomal compartment, indicating a reduced protein turnover rate or degradation. Given the direct association of ANXA2 and endocytosis, ANXA2 may be an upstream regulator of HSP90α responsible for its recycling upon stimulation. HSP90α is involved in the disruption of BBB function post cerebral ischemic stroke. Inhibition of HSP90α with ATP competitive inhibitor results in reduced activity of metalloproteinase 9 (MMP9), which plays an important role in BBB dysfunction, and rescues TJ protein expression abnormality[121]. HSP90α is also associated with inflammation induced by LPS[122]. It has been shown that the involvement of HSP90α is associated with PI3K and NF-κB pathways[123]. Therefore, a possible mechanism explaining the increased susceptibility of *ANXA2*-KO to *R*.*australis*-induced CMH is that ANXA2 depletion causes reduced degradation of HSP90α, which destabilizes the cell-cell junction and increases the hyperpermeability of BBB. So far AXNA2 is known to interact with HSP90α and such interaction is increased in the presence of high glucose[106], yet the role of HSP90α-AXNA2 interaction in bacterial infection is unknown. Further *in vitro* experiment is needed to confirm this mechanism.

HSPa8 is a noncanonical member of the heat shock protein 70 family[124]. The majority of HSPa8 resides in the cytosol and nucleus, but upon stimulation, it also translocates to the plasma membrane or exosome, contributing to antigen presentation via MHC II to CD4 T cells, clathrin-mediated vesicles transport, and chaperone-dependent autophagy[124, 125]. In our data, similar to HSP90α, HSPa8 was found to be reduced in the endosomal compartment in *ANXA2*-KO group, whereas it was upregulated in the whole brain lysate (one-fold). However, the biological role of the association between AXNA2 and HSPa8 is unknown.

HSP90α, HSP8 and mitogen-activated protein kinase kinase 4(Map2k4) comprise another functional group, stress response. Map2k4 is a direct activator of c-jun activation protein kinase (JNK) [126]. Map2k4 was enriched in the endosomal compartment in *ANXA2*-KO mice brain compared to WT. Interestingly, it has been reported that *ANXA2* knockdown by shRNA enhances the activation of JNK and p38 in response to oxidative stress[127].

### Other considerations

An earlier study reported that the deletion of ANXA2 rendered mice susceptible to chemically-induced carotid arterial thrombosis[128]. To determine the possible involvement of thrombus formation, which is associated with EC injury[129], in cerebral microhemorrhage, we examined all H&E staining but no thrombus was visualized.

We previously showed that the exchange protein directly activated by cAMP (EPAC) plays a critical role during SFGR infections[84] and that EPAC regulates ANXA2-mediated vascular fibrinolysis[130]. Given the regulatory role of EPAC on ANXA2, we retrospectively reviewed archival H&E-stained brain sections of EPAC1-deficient mice[84, 130] infected with an ordinarily lethal dose of SFGR, but no histological evidence of CMHs was found, suggesting EPAC is not associated with CMHs during infections.

### Conclusion

This is the first report that correlates the special role of ANXA2 and CMHs in the context of rickettsial and Ebola virus infections. Our data suggest that ANXA2 does not affect the in vivo proliferation of *R*. *australis* or the survival of the mice infected with *R*. *australis*. Inflammatory cytokines TNFα and IFNγ are probably not essential to ANXA2-dependent CMHs in *R*. *australis* infection. LC/MS studies using the whole brain lysates and endosomal proteins from the brain of *R*. *australis*-infected mice has revealed that deletion of ANXA2 results in a series of differentially expressed proteins that are relevant to cell-cell junction category. Consistently, histology showed that deletion of ANXA2 abrogates the structural integrity of endothelial TJs in rickettsial and Ebola virus infections, suggesting an essential role of ANXA2 in stabilization of the TJs and BBB. The detailed mechanism is unclear, but HSP90α seems to be an interesting target for future study. Further in vivo experiments are necessary to characterize the role(s) of ANXA2 in the pathogenesis of Ebola infection.

## Supporting information

S1 Fig (A) Comparing the survival of ANXA2-KO (n = 15) and WT (n = 14) mice challenged by R.australis up to 10 days. No significant difference was found based on Log-rank test. (B) Gross view of the brain surface (R. australis infected WT(top panel) & R.australis infected KO (bottom panel)). Yellow arrows indicate R. australis infected mice brains exhibt a large difference in color.
(TIF)

S2 Fig Comparing the survival of ANXA2-KO and WT mice challenged by Ebola virus up to 12 days. No significant difference was found based on Log-rank test, n = 5 for both groups, P = 0.08.
(TIF)

S1 Table GO Term CC and KEGG. Significantly different functional groups from GO Term CC and KEGG pathways are listed. LC/MS identified DE protein lists for the whole brain samples and brain derived isolated endosome samples waere incorporated into DAVID to perform the functional enrichment annotation. Fisher Eact test was used in the DAVID system to measure the statistical significance.
(XLSX)

## References

[1] Dumler JS, Walker DH. Rocky Mountain spotted fever—changing ecology and persisting virulence. N Engl J Med. 2005;353(6):551–3. 10.1056/NEJMp058138 . 16093463

[2] Rennoll SA, Rennoll-Bankert KE, Guillotte ML, Lehman SS, Driscoll TP, Beier-Sexton M, et al The Cat Flea (Ctenocephalides felis) Immune Deficiency Signaling Pathway Regulates Rickettsia typhi Infection. Infect Immun. 2018;86(1). Epub 2017/11/01. 10.1128/IAI.00562-17 29084898 PMC5736803

[3] Lamason RL, Welch MD. Actin-based motility and cell-to-cell spread of bacterial pathogens. Curr Opin Microbiol. 2017;35:48–57. Epub 2016/12/19. 10.1016/j.mib.2016.11.007 27997855 PMC5474209

[4] Parola P, Socolovschi C, Jeanjean L, Bitam I, Fournier PE, Sotto A, et al Warmer weather linked to tick attack and emergence of severe rickettsioses. PLoS Negl Trop Dis. 2008;2(11):e338 10.1371/journal.pntd.0000338 19015724 PMC2581602

[5] Riley SP, Goh KC, Hermanas TM, Cardwell MM, Chan YG, Martinez JJ. The Rickettsia conorii autotransporter protein Sca1 promotes adherence to nonphagocytic mammalian cells. Infect Immun. 2010;78(5):1895–904. 10.1128/IAI.01165-09 20176791 PMC2863548

[6] Suwanbongkot C, Langohr IM, Harris EK, Dittmar W, Christofferson RC, Macaluso KR. Spotted Fever Group. Infect Immun. 2019;87(4). Epub 2019/03/25. 10.1128/IAI.00804-18 30642897 PMC6434108

[7] Paris DH, Dumler JS. State of the art of diagnosis of rickettsial diseases: the use of blood specimens for diagnosis of scrub typhus, spotted fever group rickettsiosis, and murine typhus. Curr Opin Infect Dis. 2016;29(5):433–9. 10.1097/QCO.0000000000000298 27429138 PMC5029442

[8] Walker DH, Paddock CD, Dumler JS. Emerging and re-emerging tick-transmitted rickettsial and ehrlichial infections. Med Clin North Am. 2008;92(6):1345–61, x. 10.1016/j.mcna.2008.06.002 . 19061755

[9] Valbuena G, Walker DH. Infection of the endothelium by members of the order Rickettsiales. Thromb Haemost. 2009;102(6):1071–9. 10.1160/TH09-03-0186 19967137 PMC2913309

[10] Openshaw JJ, Swerdlow DL, Krebs JW, Holman RC, Mandel E, Harvey A, et al Rocky mountain spotted fever in the United States, 2000–2007: interpreting contemporary increases in incidence. Am J Trop Med Hyg. 2010;83(1):174–82. 10.4269/ajtmh.2010.09-0752 20595498 PMC2912596

[11] Botelho-Nevers E, Socolovschi C, Raoult D, Parola P. Treatment of Rickettsia spp. infections: a review. Expert Rev Anti Infect Ther. 2012;10(12):1425–37. 10.1586/eri.12.139 . 23253320

[12] de Sousa R, Nóbrega SD, Bacellar F, Torgal J. Mediterranean spotted fever in Portugal: risk factors for fatal outcome in 105 hospitalized patients. Ann N Y Acad Sci. 2003;990:285–94. 10.1111/j.1749-6632.2003.tb07378.x . 12860641

[13] Olejnik J, Ryabchikova E, Corley RB, Mühlberger E. Intracellular events and cell fate in filovirus infection. Viruses. 2011;3(8):1501–31. 10.3390/v3081501 21927676 PMC3172725

[14] Falzarano D, Feldmann H. Vaccines for viral hemorrhagic fevers—progress and shortcomings. Curr Opin Virol. 2013;3(3):343–51. 10.1016/j.coviro.2013.04.007 23773330 PMC3743920

[15] Feldmann H, Geisbert TW. Ebola haemorrhagic fever. Lancet. 2011;377(9768):849–62. 10.1016/S0140-6736(10)60667-8 21084112 PMC3406178

[16] Wong G, Qiu X, Olinger GG, Kobinger GP. Post-exposure therapy of filovirus infections. Trends Microbiol. 2014 10.1016/j.tim.2014.04.002 . 24794572

[17] Hartman AL, Towner JS, Nichol ST. Ebola and marburg hemorrhagic fever. Clin Lab Med. 2010;30(1):161–77. 10.1016/j.cll.2009.12.001 . 20513546

[18] Beer B, Kurth R, Bukreyev A. Characteristics of Filoviridae: Marburg and Ebola viruses. Naturwissenschaften. 1999;86(1):8–17. 10.1007/s001140050562 . 10024977

[19] Spengler JR, Prescott J, Feldmann H, Spiropoulou CF. Human immune system mouse models of Ebola virus infection. Curr Opin Virol. 2017;25:90–6. Epub 2017/08/12. 10.1016/j.coviro.2017.07.028 28810165 PMC5610641

[20] Saphire EO, Aman MJ. Feverish Quest for Ebola Immunotherapy: Straight or Cocktail? Trends Microbiol. 2016;24(9):684–6. Epub 2016/06/20. 10.1016/j.tim.2016.05.008 . 27338027

[21] Cooper TK, Huzella L, Johnson JC, Rojas O, Yellayi S, Sun MG, et al Histology, immunohistochemistry, and in situ hybridization reveal overlooked Ebola virus target tissues in the Ebola virus disease guinea pig model. Sci Rep. 2018;8(1):1250 Epub 2018/01/19. 10.1038/s41598-018-19638-x 29352230 PMC5775334

[22] Deflubé LR, Cressey TN, Hume AJ, Olejnik J, Haddock E, Feldmann F, et al Ebolavirus polymerase uses an unconventional genome replication mechanism. Proc Natl Acad Sci U S A. 2019;116(17):8535–43. Epub 2019/04/08. 10.1073/pnas.1815745116 30962389 PMC6486738

[23] Hume A, Mühlberger E. Marburg Virus Viral Protein 35 Inhibits Protein Kinase R Activation in a Cell Type-Specific Manner. J Infect Dis. 2018;218(suppl_5):S403-S8. 10.1093/infdis/jiy152 30165526 PMC6249588

[24] Johnson B, Li J, Adhikari J, Edwards MR, Zhang H, Schwarz T, et al Dimerization Controls Marburg Virus VP24-dependent Modulation of Host Antioxidative Stress Responses. J Mol Biol. 2016;428(17):3483–94. Epub 2016/08/04. 10.1016/j.jmb.2016.07.020 27497688 PMC5010500

[25] Edwards MR, Johnson B, Mire CE, Xu W, Shabman RS, Speller LN, et al The Marburg virus VP24 protein interacts with Keap1 to activate the cytoprotective antioxidant response pathway. Cell Rep. 2014;6(6):1017–25. 10.1016/j.celrep.2014.01.043 24630991 PMC3985291

[26] Vine V, Scott DP, Feldmann H. Ebolavirus: An Overview of Molecular and Clinical Pathogenesis. Methods Mol Biol. 2017;1628:39–50. 10.1007/978-1-4939-7116-9_3 . 28573609 PMC11060604

[27] Wahl-Jensen VM, Afanasieva TA, Seebach J, Ströher U, Feldmann H, Schnittler HJ. Effects of Ebola virus glycoproteins on endothelial cell activation and barrier function. J Virol. 2005;79(16):10442–50. 10.1128/JVI.79.16.10442-10450.2005 16051836 PMC1182673

[28] Wolf T, Kann G, Becker S, Stephan C, Brodt HR, de Leuw P, et al Severe Ebola virus disease with vascular leakage and multiorgan failure: treatment of a patient in intensive care. Lancet. 2015;385(9976):1428–35. Epub 2014/12/19. 10.1016/S0140-6736(14)62384-9 . 25534190

[29] Lubaki NM, Ilinykh P, Pietzsch C, Tigabu B, Freiberg AN, Koup RA, et al The lack of maturation of Ebola virus-infected dendritic cells results from the cooperative effect of at least two viral domains. J Virol. 2013;87(13):7471–85. Epub 2013/04/24. 10.1128/JVI.03316-12 23616668 PMC3700277

[30] Greenberg SM, Vernooij MW, Cordonnier C, Viswanathan A, Al-Shahi Salman R, Warach S, et al Cerebral microbleeds: a guide to detection and interpretation. Lancet Neurol. 2009;8(2):165–74. 10.1016/S1474-4422(09)70013-4 19161908 PMC3414436

[31] Ungvari Z, Tarantini S, Kirkpatrick AC, Csiszar A, Prodan CI. Cerebral microhemorrhages: mechanisms, consequences, and prevention. Am J Physiol Heart Circ Physiol. 2017;312(6):H1128–H43. Epub 2017/03/17. 10.1152/ajpheart.00780.2016 28314762 PMC5495931

[32] Schlunk F, Böhm M, Boulouis G, Qin T, Arbel M, Tamim I, et al Secondary Bleeding During Acute Experimental Intracerebral Hemorrhage. Stroke. 2019;50(5):1210–5. 10.1161/STROKEAHA.118.021732 31009358 PMC6478448

[33] Koennecke HC. Cerebral microbleeds on MRI: prevalence, associations, and potential clinical implications. Neurology. 2006;66(2):165–71. 10.1212/01.wnl.0000194266.55694.1e . 16434647

[34] Chien LN, Chi NF, Hu CJ, Chiou HY. Central nervous system infections and stroke—a population-based analysis. Acta Neurol Scand. 2013;128(4):241–8. Epub 2013/04/01. 10.1111/ane.12116 . 23550811

[35] Siler DA, Berlow YA, Kukino A, Davis CM, Nelson JW, Grafe MR, et al Soluble Epoxide Hydrolase in Hydrocephalus, Cerebral Edema, and Vascular Inflammation After Subarachnoid Hemorrhage. Stroke. 2015;46(7):1916–22. Epub 2015/05/19. 10.1161/STROKEAHA.114.008560 25991416 PMC4480190

[36] Benakis C, Garcia-Bonilla L, Iadecola C, Anrather J. The role of microglia and myeloid immune cells in acute cerebral ischemia. Front Cell Neurosci. 2014;8:461 Epub 2015/01/14. 10.3389/fncel.2014.00461 25642168 PMC4294142

[37] Baltan S. Ischemic injury to white matter: an age-dependent process. Neuroscientist. 2009;15(2):126–33. 10.1177/1073858408324788 . 19307420

[38] Guell K, Bix GJ. Brain endothelial cell specific integrins and ischemic stroke. Expert Rev Neurother. 2014;14(11):1287–92. Epub 2014/09/29. 10.1586/14737175.2014.964210 . 25262658

[39] Yang C, Hawkins KE, Doré S, Candelario-Jalil E. Neuroinflammatory mechanisms of blood-brain barrier damage in ischemic stroke. Am J Physiol Cell Physiol. 2019;316(2):C135–C53. Epub 2018/10/31. 10.1152/ajpcell.00136.2018 30379577 PMC6397344

[40] Wolf MS, Bayır H, Kochanek PM, Clark RSB. The role of autophagy in acute brain injury: A state of flux? Neurobiol Dis. 2019;122:9–15. Epub 2018/04/26. 10.1016/j.nbd.2018.04.018 29704549 PMC6203674

[41] Tomura S, de Rivero Vaccari JP, Keane RW, Bramlett HM, Dietrich WD. Effects of therapeutic hypothermia on inflammasome signaling after traumatic brain injury. J Cereb Blood Flow Metab. 2012;32(10):1939–47. Epub 2012/07/11. 10.1038/jcbfm.2012.99 22781337 PMC3463887

[42] Hsu M, Rayasam A, Kijak JA, Choi YH, Harding JS, Marcus SA, et al Neuroinflammation-induced lymphangiogenesis near the cribriform plate contributes to drainage of CNS-derived antigens and immune cells. Nat Commun. 2019;10(1):229 Epub 2019/01/16. 10.1038/s41467-018-08163-0 30651548 PMC6335416

[43] Kotoda M, Furukawa H, Miyamoto T, Korai M, Shikata F, Kuwabara A, et al Role of Myeloid Lineage Cell Autophagy in Ischemic Brain Injury. Stroke. 2018;49(6):1488–95. Epub 2018/05/10. 10.1161/STROKEAHA.117.018637 29748423 PMC5970995

[44] DeKosky ST, Abrahamson EE, Ciallella JR, Paljug WR, Wisniewski SR, Clark RS, et al Association of increased cortical soluble abeta42 levels with diffuse plaques after severe brain injury in humans. Arch Neurol. 2007;64(4):541–4. 10.1001/archneur.64.4.541 . 17420316

[45] Nasr IW, Chun Y, Kannan S. Neuroimmune responses in the developing brain following traumatic brain injury. Exp Neurol. 2019;320:112957 Epub 2019/05/17. 10.1016/j.expneurol.2019.112957 . 31108085

[46] Su EJ, Lawrence DA. α2 Antiplasmin and microvascular thrombosis in ischemic stroke. Arterioscler Thromb Vasc Biol. 2014;34(12):2522–3. 10.1161/ATVBAHA.114.304616 . 25411105

[47] Sabirzhanov B, Faden AI, Aubrecht T, Henry R, Glaser E, Stoica BA. MicroRNA-711-Induced Downregulation of Angiopoietin-1 Mediates Neuronal Cell Death. J Neurotrauma. 2018;35(20):2462–81. Epub 2018/07/10. 10.1089/neu.2017.5572 29774773 PMC6196751

[48] Yi JH, Park SW, Kapadia R, Vemuganti R. Role of transcription factors in mediating post-ischemic cerebral inflammation and brain damage. Neurochem Int. 2007;50(7–8):1014–27. Epub 2007/05/03. 10.1016/j.neuint.2007.04.019 17532542 PMC2040388

[49] Ding Y, Flores J, Klebe D, Li P, McBride DW, Tang J, et al Annexin A1 attenuates neuroinflammation through FPR2/p38/COX-2 pathway after intracerebral hemorrhage in male mice. J Neurosci Res. 2019 Epub 2019/06/03. 10.1002/jnr.24478 . 31157469 PMC6854313

[50] Askenase MH, Sansing LH. Stages of the Inflammatory Response in Pathology and Tissue Repair after Intracerebral Hemorrhage. Semin Neurol. 2016;36(3):288–97. Epub 2016/05/23. 10.1055/s-0036-1582132 27214704 PMC4956485

[51] Han X, Lan X, Li Q, Gao Y, Zhu W, Cheng T, et al Inhibition of prostaglandin E2 receptor EP3 mitigates thrombin-induced brain injury. J Cereb Blood Flow Metab. 2016;36(6):1059–74. Epub 2015/10/02. 10.1177/0271678X15606462 26661165 PMC4908617

[52] Bonsack F, Alleyne CH, Sukumari-Ramesh S. Augmented expression of TSPO after intracerebral hemorrhage: a role in inflammation? J Neuroinflammation. 2016;13(1):151 Epub 2016/06/17. 10.1186/s12974-016-0619-2 27315802 PMC4912814

[53] Jickling GC, Ander BP, Shroff N, Orantia M, Stamova B, Dykstra-Aiello C, et al Leukocyte response is regulated by microRNA let7i in patients with acute ischemic stroke. Neurology. 2016;87(21):2198–205. Epub 2016/10/26. 10.1212/WNL.0000000000003354 27784773 PMC5123554

[54] Chen-Roetling J, Cao Y, Peng D, Regan RF. Rapid loss of perihematomal cell viability in the collagenase intracerebral hemorrhage model. Brain Res. 2019;1711:91–6. Epub 2019/01/10. 10.1016/j.brainres.2019.01.014 30639124 PMC6519080

[55] Huggins MA, Johnson HL, Jin F, N Songo A, Hanson LM, LaFrance SJ, et al Perforin Expression by CD8 T Cells Is Sufficient To Cause Fatal Brain Edema during Experimental Cerebral Malaria. Infect Immun. 2017;85(5). Epub 2017/04/21. 10.1128/IAI.00985-16 28264905 PMC5400849

[56] Willenbring RC, Jin F, Hinton DJ, Hansen M, Choi DS, Pavelko KD, et al Modulatory effects of perforin gene dosage on pathogen-associated blood-brain barrier (BBB) disruption. J Neuroinflammation. 2016;13(1):222 Epub 2016/08/31. 10.1186/s12974-016-0673-9 27576583 PMC5006384

[57] Yang D, Sun YY, Lin X, Baumann JM, Dunn RS, Lindquist DM, et al Intranasal delivery of cell-penetrating anti-NF-κB peptides (Tat-NBD) alleviates infection-sensitized hypoxic-ischemic brain injury. Exp Neurol. 2013;247:447–55. Epub 2013/01/23. 10.1016/j.expneurol.2013.01.015 23353638 PMC4064308

[58] Aronowski J, Roy-O'Reilly MA. Neutrophils, the Felons of the Brain. Stroke. 2019;50(3):e42–e3. 10.1161/STROKEAHA.118.021563 30674235 PMC6544162

[59] Manousakis G, Jensen MB, Chacon MR, Sattin JA, Levine RL. The interface between stroke and infectious disease: infectious diseases leading to stroke and infections complicating stroke. Curr Neurol Neurosci Rep. 2009;9(1):28–34. 10.1007/s11910-009-0005-x . 19080750

[60] Nakano K, Hokamura K, Taniguchi N, Wada K, Kudo C, Nomura R, et al The collagen-binding protein of Streptococcus mutans is involved in haemorrhagic stroke. Nat Commun. 2011;2:485 Epub 2011/09/27. 10.1038/ncomms1491 21952219 PMC3220351

[61] Hyacinth HI, Sugihara CL, Spencer TL, Archer DR, Shih AY. Higher prevalence of spontaneous cerebral vasculopathy and cerebral infarcts in a mouse model of sickle cell disease. J Cereb Blood Flow Metab. 2019;39(2):342–51. Epub 2017/09/19. 10.1177/0271678X17732275 28925802 PMC6365608

[62] Corrêa DG, Cruz Júnior LC, Bahia PR, Gasparetto EL. Intracerebral microbleeds in sepsis: susceptibility-weighted MR imaging findings. Arq Neuropsiquiatr. 2012;70(11):903–4. 10.1590/s0004-282x2012001100017 . 23175208

[63] Amaro M, Bacellar F, França A. Report of eight cases of fatal and severe Mediterranean spotted fever in Portugal. Ann N Y Acad Sci. 2003;990:331–43. 10.1111/j.1749-6632.2003.tb07384.x . 12860647

[64] Horney LF, Walker DH. Meningoencephalitis as a major manifestation of Rocky Mountain spotted fever. South Med J. 1988;81(7):915–8. 10.1097/00007611-198807000-00028 . 3393952

[65] Garc Ía-Baena C, Cárdenas MF, Ramón JF. Cerebral haemorrhage as a clinical manifestation of human ehrlichiosis. BMJ Case Rep. 2017;2017 Epub 2017/07/27. 10.1136/bcr-2016-219054 . 28751428 PMC5612296

[66] Bonney S, Seitz S, Ryan CA, Jones KL, Clarke P, Tyler KL, et al Gamma Interferon Alters Junctional Integrity via Rho Kinase, Resulting in Blood-Brain Barrier Leakage in Experimental Viral Encephalitis. MBio. 2019;10(4). Epub 2019/08/06. 10.1128/mBio.01675-19 31387911 PMC6686045

[67] Sumbria RK, Grigoryan MM, Vasilevko V, Krasieva TB, Scadeng M, Dvornikova AK, et al A murine model of inflammation-induced cerebral microbleeds. J Neuroinflammation. 2016;13(1):218 Epub 2016/08/30. 10.1186/s12974-016-0693-5 27577728 PMC5006574

[68] Joó F. The blood-brain barrier. Nature. 1987;329(6136):208 10.1038/329208b0 . 3627266

[69] Zhao Z, Nelson AR, Betsholtz C, Zlokovic BV. Establishment and Dysfunction of the Blood-Brain Barrier. Cell. 2015;163(5):1064–78. 10.1016/j.cell.2015.10.067 26590417 PMC4655822

[70] Aydin F, Rosenblum WI, Povlishock JT. Myoendothelial junctions in human brain arterioles. Stroke. 1991;22(12):1592–7. 10.1161/01.str.22.12.1592 . 1962335

[71] Chen B, Friedman B, Cheng Q, Tsai P, Schim E, Kleinfeld D, et al Severe blood-brain barrier disruption and surrounding tissue injury. Stroke. 2009;40(12):e666–74. Epub 2009/11/05. 10.1161/STROKEAHA.109.551341 19893002 PMC2819286

[72] Ramirez SH, Andrews AM, Paul D, Pachter JS. Extracellular vesicles: mediators and biomarkers of pathology along CNS barriers. Fluids Barriers CNS. 2018;15(1):19 Epub 2018/07/01. 10.1186/s12987-018-0104-7 29960602 PMC6026502

[73] Hayes MJ, Shao D, Bailly M, Moss SE. Regulation of actin dynamics by annexin 2. EMBO J. 2006;25(9):1816–26. Epub 2006/04/08. 10.1038/sj.emboj.7601078 16601677 PMC1456940

[74] Liu Y, Myrvang HK, Dekker LV. Annexin A2 complexes with S100 proteins: structure, function and pharmacological manipulation. Br J Pharmacol. 2014 10.1111/bph.12978 . 25303710 PMC4376447

[75] Bharadwaj A, Bydoun M, Holloway R, Waisman D. Annexin A2 heterotetramer: structure and function. Int J Mol Sci. 2013;14(3):6259–305. 10.3390/ijms14036259 23519104 PMC3634455

[76] Grieve AG, Moss SE, Hayes MJ. Annexin A2 at the interface of actin and membrane dynamics: a focus on its roles in endocytosis and cell polarization. Int J Cell Biol. 2012;2012:852430 Epub 2012/04/17. 10.1155/2012/852430 22505935 PMC3296266

[77] Rescher U, Ludwig C, Konietzko V, Kharitonenkov A, Gerke V. Tyrosine phosphorylation of annexin A2 regulates Rho-mediated actin rearrangement and cell adhesion. J Cell Sci. 2008;121(Pt 13):2177–85. 10.1242/jcs.028415 . 18565825

[78] Garrido-Gómez T, Dominguez F, Quiñonero A, Estella C, Vilella F, Pellicer A, et al Annexin A2 is critical for embryo adhesiveness to the human endometrium by RhoA activation through F-actin regulation. FASEB J. 2012;26(9):3715–27. 10.1096/fj.12-204008 . 22645245

[79] Jung Y, Wang J, Song J, Shiozawa Y, Havens A, Wang Z, et al Annexin II expressed by osteoblasts and endothelial cells regulates stem cell adhesion, homing, and engraftment following transplantation. Blood. 2007;110(1):82–90. 10.1182/blood-2006-05-021352 17360942 PMC1896130

[80] Luo M, Hajjar KA. Annexin A2 system in human biology: cell surface and beyond. Semin Thromb Hemost. 2013;39(4):338–46. 10.1055/s-0033-1334143 23483454 PMC3869233

[81] Hajjar KA. The Biology of Annexin A2: From Vascular Fibrinolysis to Innate Immunity. Trans Am Clin Climatol Assoc. 2015;126:144–55. 26330668 PMC4530673

[82] He X, Zhang W, Chang Q, Su Z, Gong D, Zhou Y, et al A new role for host annexin A2 in establishing bacterial adhesion to vascular endothelial cells. Laboratory Investigation. 2019. 10.1038/s41374-019-0284-z PMC6913097 31253864

[83] Gong B, Lee YS, Lee I, Shelite TR, Kunkeaw N, Xu G, et al Compartmentalized, functional role of angiogenin during spotted fever group rickettsia-induced endothelial barrier dysfunction: evidence of possible mediation by host tRNA-derived small noncoding RNAs. BMC Infect Dis. 2013;13:285 10.1186/1471-2334-13-285 23800282 PMC3699377

[84] Gong B, Shelite T, Mei FC, Ha T, Hu Y, Xu G, et al Exchange protein directly activated by cAMP plays a critical role in bacterial invasion during fatal rickettsioses. Proc Natl Acad Sci U S A. 2013;110(48):19615–20. 10.1073/pnas.1314400110 24218580 PMC3845138

[85] Bray M, Davis K, Geisbert T, Schmaljohn C, Huggins J. A mouse model for evaluation of prophylaxis and therapy of Ebola hemorrhagic fever. J Infect Dis. 1998;178(3):651–61. 10.1086/515386 . 9728532

[86] Smalley C, Bechelli J, Rockx-Brouwer D, Saito T, Azar SR, Ismail N, et al Rickettsia australis Activates Inflammasome in Human and Murine Macrophages. PLoS One. 2016;11(6):e0157231 Epub 2016/07/01. 10.1371/journal.pone.0157231 27362650 PMC4928923

[87] Manadas B, English JA, Wynne KJ, Cotter DR, Dunn MJ. Comparative analysis of OFFGel, strong cation exchange with pH gradient, and RP at high pH for first-dimensional separation of peptides from a membrane-enriched protein fraction. Proteomics. 2009;9(22):5194–8. 10.1002/pmic.200900349 . 19771557

[88] Sowers ML, Re JD, Wadsworth PA, Shavkunov AS, Lichti C, Zhang K, et al Sex-Specific Proteomic Changes Induced by Genetic Deletion of Fibroblast Growth Factor 14 (FGF14), a Regulator of Neuronal Ion Channels. Proteomes. 2019;7(1). Epub 2019/01/23. 10.3390/proteomes7010005 30678040 PMC6473632

[89] Nesvizhskii AI, Keller A, Kolker E, Aebersold R. A statistical model for identifying proteins by tandem mass spectrometry. Anal Chem. 2003;75(17):4646–58. Epub 2003/11/25. 10.1021/ac0341261 . 14632076

[90] Maere S, Heymans K, Kuiper M. BiNGO: a Cytoscape plugin to assess overrepresentation of gene ontology categories in biological networks. Bioinformatics. 2005;21(16):3448–9. Epub 2005/06/24. 10.1093/bioinformatics/bti551 . 15972284

[91] Mostafavi S, Ray D, Warde-Farley D, Grouios C, Morris Q. GeneMANIA: a real-time multiple association network integration algorithm for predicting gene function. Genome Biol. 2008;9 Suppl 1:S4 Epub 2008/07/22. 10.1186/gb-2008-9-s1-s4 18613948 PMC2447538

[92] Lu H, Sun TX, Bouley R, Blackburn K, McLaughlin M, Brown D. Inhibition of endocytosis causes phosphorylation (S256)-independent plasma membrane accumulation of AQP2. Am J Physiol Renal Physiol. 2004;286(2):F233–43. Epub 2003/10/02. 10.1152/ajprenal.00179.2003 . 14519593

[93] Lokman NA, Ween MP, Oehler MK, Ricciardelli C. The role of annexin A2 in tumorigenesis and cancer progression. Cancer Microenviron. 2011;4(2):199–208. Epub 2011/09/13. 10.1007/s12307-011-0064-9 21909879 PMC3170418

[94] Wang S, Sun H, Tanowitz M, Liang XH, Crooke ST. Annexin A2 facilitates endocytic trafficking of antisense oligonucleotides. Nucleic Acids Res. 2016;44(15):7314–30. Epub 2016/07/06. 10.1093/nar/gkw595 27378781 PMC5009748

[95] Zhang S, Yu M, Guo Q, Li R, Li G, Tan S, et al Annexin A2 binds to endosomes and negatively regulates TLR4-triggered inflammatory responses via the TRAM-TRIF pathway. Sci Rep. 2015;5:15859 Epub 2015/11/04. 10.1038/srep15859 26527544 PMC4630631

[96] Law AL, Ling Q, Hajjar KA, Futter CE, Greenwood J, Adamson P, et al Annexin A2 regulates phagocytosis of photoreceptor outer segments in the mouse retina. Mol Biol Cell. 2009;20(17):3896–904. Epub 2009/07/10. 10.1091/mbc.e08-12-1204 19587120 PMC2735488

[97] Morozova K, Sridhar S, Zolla V, Clement CC, Scharf B, Verzani Z, et al Annexin A2 promotes phagophore assembly by enhancing Atg16L(+) vesicle biogenesis and homotypic fusion. Nat Commun. 2015;6:5856 Epub 2015/01/20. 10.1038/ncomms6856 25597631 PMC4299943

[98] Rentero C, Blanco-Munoz P, Meneses-Salas E, Grewal T, Enrich C. Annexins-Coordinators of Cholesterol Homeostasis in Endocytic Pathways. Int J Mol Sci. 2018;19(5). Epub 2018/05/15. 10.3390/ijms19051444 29757220 PMC5983649

[99] Maiers JL, Peng X, Fanning AS, DeMali KA. ZO-1 recruitment to alpha-catenin—a novel mechanism for coupling the assembly of tight junctions to adherens junctions. J Cell Sci. 2013;126(Pt 17):3904–15. Epub 2013/07/03. 10.1242/jcs.126565 23813953 PMC3757330

[100] Martines RB, Ng DL, Greer PW, Rollin PE, Zaki SR. Tissue and cellular tropism, pathology and pathogenesis of Ebola and Marburg viruses. J Pathol. 2015;235(2):153–74. Epub 2014/10/10. 10.1002/path.4456 . 25297522

[101] Campbell HK, Maiers JL, DeMali KA. Interplay between tight junctions & adherens junctions. Exp Cell Res. 2017;358(1):39–44. Epub 2017/03/31. 10.1016/j.yexcr.2017.03.061 28372972 PMC5544570

[102] Antonov A, Snead C, Gorshkov B, Antonova GN, Verin AD, Catravas JD. Heat shock protein 90 inhibitors protect and restore pulmonary endothelial barrier function. Am J Respir Cell Mol Biol. 2008;39(5):551–9. Epub 2008/05/14. 10.1165/rcmb.2007-0324OC 18474672 PMC2574526

[103] Chatterjee A, Snead C, Yetik-Anacak G, Antonova G, Zeng J, Catravas JD. Heat shock protein 90 inhibitors attenuate LPS-induced endothelial hyperpermeability. Am J Physiol Lung Cell Mol Physiol. 2008;294(4):L755–63. Epub 2008/02/05. 10.1152/ajplung.00350.2007 . 18245267

[104] Joshi AD, Dimitropoulou C, Thangjam G, Snead C, Feldman S, Barabutis N, et al Heat shock protein 90 inhibitors prevent LPS-induced endothelial barrier dysfunction by disrupting RhoA signaling. Am J Respir Cell Mol Biol. 2014;50(1):170–9. Epub 2013/08/27. 10.1165/rcmb.2012-0496OC 23972231 PMC3930930

[105] Stenos J, Walker DH. The rickettsial outer-membrane protein A and B genes of Rickettsia australis, the most divergent rickettsia of the spotted fever group. Int J Syst Evol Microbiol. 2000;50 Pt 5:1775–9. Epub 2000/10/18. 10.1099/00207713-50-5-1775 . 11034486

[106] Lei H, Romeo G, Kazlauskas A. Heat shock protein 90alpha-dependent translocation of annexin II to the surface of endothelial cells modulates plasmin activity in the diabetic rat aorta. Circ Res. 2004;94(7):902–9. Epub 2004/03/06. 10.1161/01.RES.0000124979.46214.E3 . 15001530

[107] Tojkander S, Gateva G, Lappalainen P. Actin stress fibers—assembly, dynamics and biological roles. J Cell Sci. 2012;125(Pt 8):1855–64. Epub 2012/05/01. 10.1242/jcs.098087 . 22544950

[108] Colombelli J, Besser A, Kress H, Reynaud EG, Girard P, Caussinus E, et al Mechanosensing in actin stress fibers revealed by a close correlation between force and protein localization. J Cell Sci. 2009;122(Pt 10):1665–79. Epub 2009/04/30. 10.1242/jcs.042986 . 19401336

[109] Hoelzle MK, Svitkina T. The cytoskeletal mechanisms of cell-cell junction formation in endothelial cells. Mol Biol Cell. 2012;23(2):310–23. Epub 2011/11/18. 10.1091/mbc.E11-08-0719 22090347 PMC3258175

[110] Tinsley JH, Wu MH, Ma W, Taulman AC, Yuan SY. Activated neutrophils induce hyperpermeability and phosphorylation of adherens junction proteins in coronary venular endothelial cells. J Biol Chem. 1999;274(35):24930–4. Epub 1999/08/24. 10.1074/jbc.274.35.24930 . 10455168

[111] Sun H, Breslin JW, Zhu J, Yuan SY, Wu MH. Rho and ROCK signaling in VEGF-induced microvascular endothelial hyperpermeability. Microcirculation. 2006;13(3):237–47. Epub 2006/04/22. 10.1080/10739680600556944 . 16627366

[112] Bauer HC, Krizbai IA, Bauer H, Traweger A. "You Shall Not Pass"-tight junctions of the blood brain barrier. Front Neurosci. 2014;8:392 Epub 2014/12/03. 10.3389/fnins.2014.00392 25520612 PMC4253952

[113] Fanning AS, Jameson BJ, Jesaitis LA, Anderson JM. The tight junction protein ZO-1 establishes a link between the transmembrane protein occludin and the actin cytoskeleton. J Biol Chem. 1998;273(45):29745–53. Epub 1998/10/29. 10.1074/jbc.273.45.29745 . 9792688

[114] Lai CH, Kuo KH, Leo JM. Critical role of actin in modulating BBB permeability. Brain Res Brain Res Rev. 2005;50(1):7–13. Epub 2005/11/18. 10.1016/j.brainresrev.2005.03.007 . 16291072

[115] Lum H, Roebuck KA. Oxidant stress and endothelial cell dysfunction. Am J Physiol Cell Physiol. 2001;280(4):C719–41. Epub 2001/03/14. 10.1152/ajpcell.2001.280.4.C719 . 11245588

[116] Georgiou M, Marinari E, Burden J, Baum B. Cdc42, Par6, and aPKC regulate Arp2/3-mediated endocytosis to control local adherens junction stability. Curr Biol. 2008;18(21):1631–8. Epub 2008/11/04. 10.1016/j.cub.2008.09.029 . 18976918

[117] Gerhardt H, Wolburg H, Redies C. N-cadherin mediates pericytic-endothelial interaction during brain angiogenesis in the chicken. Dev Dyn. 2000;218(3):472–9. Epub 2000/07/06. 10.1002/1097-0177(200007)218:3&lt;472::AID-DVDY1008&gt;3.0.CO;2-%23 . 10878612

[118] Fiorini C, Gilleron J, Carette D, Valette A, Tilloy A, Chevalier S, et al Accelerated internalization of junctional membrane proteins (connexin 43, N-cadherin and ZO-1) within endocytic vacuoles: an early event of DDT carcinogenicity. Biochim Biophys Acta. 2008;1778(1):56–67. Epub 2007/10/24. 10.1016/j.bbamem.2007.08.032 . 17949680

[119] Eddinger TJ, Schiebout JD, Swartz DR. Adherens junction-associated protein distribution differs in smooth muscle tissue and acutely isolated cells. Am J Physiol Gastrointest Liver Physiol. 2007;292(2):G684–97. Epub 2006/10/21. 10.1152/ajpgi.00277.2006 . 17053160

[120] Alanko J, Ivaska J. Endosomes: Emerging Platforms for Integrin-Mediated FAK Signalling. Trends Cell Biol. 2016;26(6):391–8. Epub 2016/03/06. 10.1016/j.tcb.2016.02.001 . 26944773

[121] Qi J, Liu Y, Yang P, Chen T, Liu XZ, Yin Y, et al Heat shock protein 90 inhibition by 17-Dimethylaminoethylamino-17-demethoxygeldanamycin protects blood-brain barrier integrity in cerebral ischemic stroke. Am J Transl Res. 2015;7(10):1826–37. Epub 2015/12/23. 26692927 PMC4656760

[122] Poulaki V, Iliaki E, Mitsiades N, Mitsiades CS, Paulus YN, Bula DV, et al Inhibition of Hsp90 attenuates inflammation in endotoxin-induced uveitis. FASEB J. 2007;21(9):2113–23. Epub 2007/04/03. 10.1096/fj.06-7637com . 17400913

[123] Shimp SK 3rd, Parson CD, Regna NL, Thomas AN, Chafin CB, Reilly CM, et al HSP90 inhibition by 17-DMAG reduces inflammation in J774 macrophages through suppression of Akt and nuclear factor-kappaB pathways. Inflamm Res. 2012;61(5):521–33. Epub 2012/02/14. 10.1007/s00011-012-0442-x . 22327510

[124] Akhter S, Chakraborty S, Moutinho D, Alvarez-Coiradas E, Rosa I, Vinuela J, et al The human VGF-derived bioactive peptide TLQP-21 binds heat shock 71 kDa protein 8 (HSPA8)on the surface of SH-SY5Y cells. PLoS One. 2017;12(9):e0185176 Epub 2017/09/22. 10.1371/journal.pone.0185176 28934328 PMC5608341

[125] Bonam SR, Ruff M, Muller S. HSPA8/HSC70 in Immune Disorders: A Molecular Rheostat that Adjusts Chaperone-Mediated Autophagy Substrates. Cells. 2019;8(8). Epub 2019/08/10. 10.3390/cells8080849 . 31394830 PMC6721745

[126] Kyriakis JM, Avruch J. Mammalian MAPK signal transduction pathways activated by stress and inflammation: a 10-year update. Physiol Rev. 2012;92(2):689–737. Epub 2012/04/27. 10.1152/physrev.00028.2011 . 22535895

[127] Madureira PA, Hill R, Miller VA, Giacomantonio C, Lee PW, Waisman DM. Annexin A2 is a novel cellular redox regulatory protein involved in tumorigenesis. Oncotarget. 2011;2(12):1075–93. Epub 2011/12/22. 10.18632/oncotarget.375 22185818 PMC3282068

[128] Ling Q, Jacovina AT, Deora A, Febbraio M, Simantov R, Silverstein RL, et al Annexin II regulates fibrin homeostasis and neoangiogenesis in vivo. J Clin Invest. 2004;113(1):38–48. 10.1172/JCI19684 14702107 PMC300771

[129] Libby P. Once more unto the breach: endothelial permeability and atherogenesis. Eur Heart J. 2019;40(11):938–40. Epub 2019/02/26. 10.1093/eurheartj/ehz081 . 30805590

[130] He X, Drelich A, Yu S, Chang Q, Gong D, Zhou Y, et al Exchange protein directly activated by cAMP plays a critical role in regulation of vascular fibrinolysis. Life Sci. 2019;221:1–12. Epub 2019/02/07. 10.1016/j.lfs.2019.02.014 . 30738042 PMC6918504

