# Supplementary figures and images for: Annexin A2 depletion exacerbates the intracerebral microhemorrhage induced by acute rickettsia and Ebola virus infections
Source: PLoS Negl Trop Dis. 2020 Jul 20;14(7):e0007960. doi: 10.1371/journal.pntd.0007960 (PMC7392349; doi:10.1371/journal.pntd.0007960)

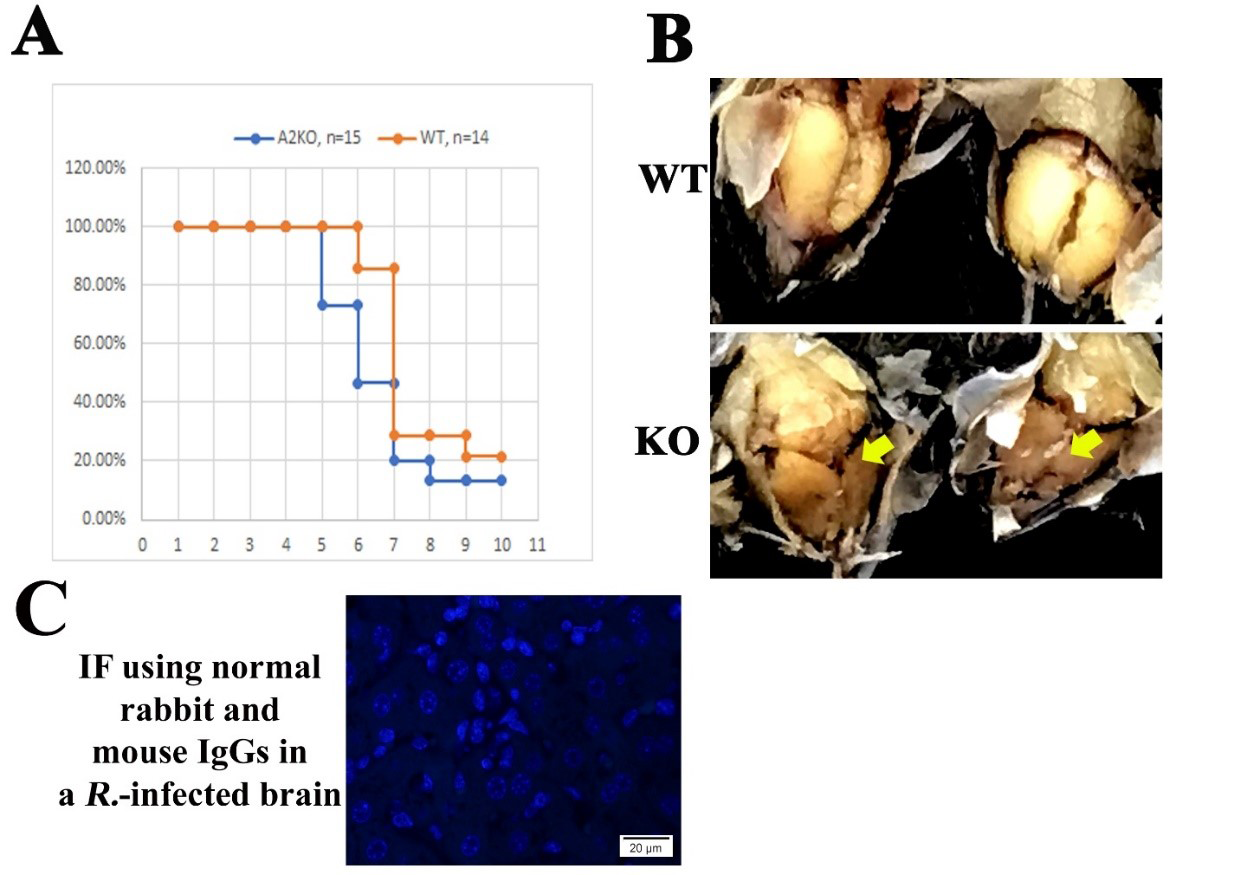

Supplement: S1 Fig — (A) Comparing the survival of ANXA2-KO (n = 15) and WT (n = 14) mice challenged by R.australis up to 10 days. No significant difference was found based on Log-rank test. (B) Gross view of the brain surface (R. australis infected WT(top panel) & R.australis infected KO (bottom panel)). Yellow arrows indicate R. australis infected mice brains exhibt a large difference in color. (TIF) [file pntd.0007960.s001.tif]

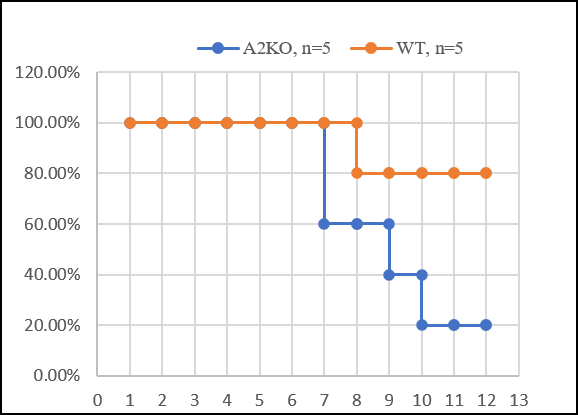

Supplement: S2 Fig — No significant difference was found based on Log-rank test, n = 5 for both groups, P = 0.08. (TIF) [file pntd.0007960.s002.tif]
